# Evolution of Repetitive Genomic Content and Gene Families Over Geo‐Climatic Gradients in Brassicaceae

**DOI:** 10.1002/ece3.72462

**Published:** 2025-12-09

**Authors:** Jana M. Flury, Weihong Qi, Olivier Bachmann, Yvonne Willi

**Affiliations:** ^1^ Department of Environmental Sciences University of Basel Basel Switzerland; ^2^ Functional Genomics Center Zurich ETH Zurich and University of Zurich Zurich Switzerland; ^3^ SIB Swiss Institute of Bioinformatics Geneva Switzerland

**Keywords:** comparative genomics, elevation, gene family expansion and contraction, latitudinal gradient, parallel evolution, transposable elements

## Abstract

On temperature gradients such as elevation or latitude, species turnover is common, and specialists can persist in extreme environments. This is likely paralleled by adaptive and possibly also non‐adaptive changes on a molecular level, from genes to the structure of genomes. Here, we investigated associations between elevation and latitude, partly represented by climate variables, with features of the genome, including genome size, transposable element (TE) content, and gene family expansion and contraction in the plant family Brassicaceae. Together, the geo‐climatic variables were good predictors of genome size and TE content, explaining 40%–60% of the variation among species. The relationship of TE content with mean annual temperature was U‐shaped, with species of cooler and hotter climates generally having more TEs, and those with elevation and mean annual precipitation (both corrected for temperature) were positive. Patterns were most prevalent for the most abundant TE class, long terminal repeat elements (LTR). Gene family expansions and contractions in species of high elevations highlighted a restructured genomic architecture of cell wall modeling, response to temperature stimulus, and processes involved in posttranslational protein modifications. Results point to abiotically extreme environments either promoting high TE content or constraining TE elimination at the level of species. Furthermore, establishing in distinct geo‐climatic regions seems associated with considerable parallel evolution with overlapping gene families changing copy numbers.

## Graphical abstract

Along temperature gradients such as elevation and latitude, species typically occupy distinct ranges, and life at the extremes may come with adaptive and non‐adaptive genomic changes. In the Brassicaceae family, comparative genomics revealed that geo‐climatic factors strongly predict genome size and transposable element (TE) content, explaining up to 60% of interspecific variation, with a U‐shaped relationship between temperature and TE abundance. Also, high‐elevation species showed notable increases in DNA‐TEs and changes in gene family sizes linked to cell wall remodeling, temperature response, and protein modification. Results suggest that extreme environments affect TE dynamics and drive parallel genomic evolution.

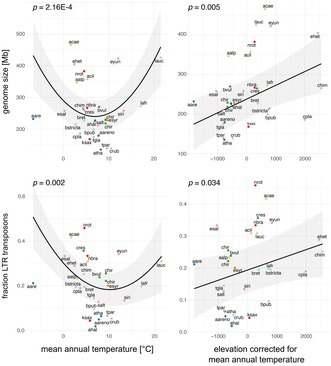

## 1 Introduction

Environmental gradients over elevation and latitude can serve as natural laboratories for studying adaptation and speciation. These gradients align with changes in several abiotic factors, presenting numerous challenges to species, particularly at their extremes. One common factor is declining temperature, with its many facets, of generally cooler conditions and possibly a snow cover during part of the year that shortens the growing season (Billings 1974; Billings and Bliss 1959). High‐elevation and high‐latitude species have therefore evolved adaptations to cope with these environmental aspects (Alonso‐Amelot 2008; Crawford 2004; Körner 2016). At the same time, the species that have evolved to live in cold environments have often experienced population genetic difficulties such as genetic drift or inbreeding (Angert et al. 2020). Other studies suggest a difference in molecular evolutionary rates between warm and cold environments, with a decreased mutation rate and lower mutational robustness at cold temperatures (Puurtinen et al. 2016). All these factors could have left an imprint on parts of the genome, the composition of genomes, and genome size.

Elevational and latitudinal gradients have ubiquitous abiotic features, and species typically occupy only a small fraction of these gradients. At higher elevations, solar radiation and wind exposure intensify, temperatures are colder and are often below freezing, there are more days with snow cover, and the reduced atmospheric pressure leads to diminished carbon dioxide levels (Körner 2003; Tranquillini 1964). Several abiotic factors change in a similar way on a latitudinal gradient. For example, temperatures get lower toward the poles, and the number of days with snow cover increases. Beyond that, at higher latitudes, the sun angle is lower and day length varies more strongly over the year (Wielgolaski and Inouye 2003). Because species occurring along such gradients are often specialized to a limited range, this results in species turnover along the gradient. Elevational gradients show this pattern globally, with well‐documented examples including birds in Colombia (Caro et al. 2013), members of the ginger family in the Himalayas (Zhao et al. 2021), or orchids on Réunion island (Jacquemyn et al. 2007). For latitudinal gradients, a famous example is bats (Willig and Gannon 1997), but the same pattern has been shown in a variety of larger taxa on different continents (Koleff et al. 2003).

In regard to phenotypic changes, adaptation to elevation involves a suite of strategies geared towards optimizing resource acquisition and/or utilization, and improving stress tolerance. For example, in herbaceous plants, alpine taxa are generally small and slow‐growing; because of the shorter growing season, they have limited time to reach the reproductive stage, and being closer to the ground means more warmth and protection against wind (Körner 2003; Maccagni and Willi 2022; Pellissier et al. 2010). Further, alpine plants tend to have smaller but thicker leaves; however there are also contrasting results involving increased leaf area (Li and Bao 2014; Zhang et al. 2010; Zhong et al. 2014) and decreased leaf thickness with higher elevation (Li and Bao 2014; Liu et al. 2018; Sun et al. 2016; Tang et al. 2015). The same is true for stomatal density; stomatal density was found to significantly increase with higher elevation in six species of the fern genus *Elaphoglossum* from the Bolivian Andes, but there was no significant increase in the other three tested species of the same genus (Kessler et al. 2007). However, in a study of a mixture of 12 species from the Southern Alps in New Zealand, a decrease in stomatal density with increasing elevation was found (Körner et al. 1986). This illustrates the complexity of plant adaptation to high elevation and that some traits show parallelism among species, whereas other species evolve divergent ways to respond to these extreme environments.

Genome screens have confirmed structural changes in genes with elevation. Some studies looked at whether particular genes or gene families revealed patterns aligning with elevation. In a comparison of two *Eutrema* (Brassiaceae) species, the one occurring at higher elevations showed duplicated genes and gene family expansions involved in disease resistance, DNA repair, reproduction, and cold tolerance (Guo et al. 2018). Several other studies found either expanded gene families, genes associated with selective sweeps or rapidly evolving genes related to DNA repair, supposedly as a response to greater potential of DNA damage because of higher levels of UV radiation at higher elevations (Chen et al. 2019; Li et al. 2021; Yang et al. 2020). In a comparative genomic study on 20 species of seven different plant families, 36 convergently selected genes in at least three among the seven alpine species were found (Zhang et al. 2023). These genes were related to reproduction, respiration, and photorespiration. Results suggest that adaptation to elevation—at least in part—involves the same genes across large phylogenetic distances and therefore conforms with some convergent evolution.

Other convergent evolution on the genome level may involve repetitive elements. It has been shown that stressful environments such as low temperatures can have an effect on transposable element (TE) activity in angiosperms (Casacuberta and González 2013; Zeh et al. 2009). Cellular TE repression mechanisms are sensitive to perturbation and stress (Oliver et al. 2013), which would explain TE expansion in species at higher elevations or latitudes. Although such TE expansion may be predominantly neutral to slightly deleterious, it could also be of partial advantage. A study on seed beetles revealed that a larger genome size because of TE expansion helped buffer adult fitness against environmental stress (Boman and Arnqvist 2023). In plants, genome size is closely associated with TE abundance (Bennett and Leitch 2005; Tenaillon et al. 2010), although a relationship with climate has rarely been explored. Such a connection could either be direct, or indirect—for example, through the mating system, since colonization of cooler environments may coincide with shifts towards selfing, which reduces the efficacy of TE elimination (Bonchev and Willi 2018; Charlesworth and Charlesworth 1995).

We studied genomic change linked to geo‐climatic gradients within the plant family Brassicaceae. We restricted ourselves to this taxonomic resolution to accommodate the trade‐off in studying parallel or convergent evolution on different phylogenetic levels; the higher the level, the less likely it becomes to find convergence (Conte et al. 2012). The Brassicaceae is a large angiosperm family comprised of ca. 350 genera and nearly 4000 species (Kiefer et al. 2014; Walden et al. 2020). There are three major lineages (I, II, and III) or six major clades within the core Brassicaceae (Beilstein et al. 2008; Guo et al. 2017; Huang et al. 2016; Nikolov et al. 2019). The family has many species and entire genera that occur at high elevations, in mountain massifs predominantly of the northern hemisphere, but also in South America (Salariato et al. 2016) and Australia/New Zealand (Hewson 1981; Mummenhoff et al. 2004). For this study, we had seven diploid species of the Brassicaceae family newly sequenced, three with a predominantly low‐elevation distribution and three with a high‐elevation distribution in the Alps, and one species with a distribution in the northern‐hemisphere subarctic and arctic. Additionally, we included haploid reference genomes of 35 other Brassicaceae species. We tested whether their elevational and latitudinal distributions were correlated with the abundance of TEs and genome size, the latter computationally estimated by *k*‐mer frequencies (Li and Waterman 2003), providing a more accurate measure of genome size compared to assembly size (e.g., Sun et al. 2018). Latitude was approximated with mean annual temperature, and mean annual precipitation was included in the analysis to represent another aspect of average climate. Furthermore, we investigated convergence in expansion and contraction in gene families among species, likely facilitating adaptation to environmental challenges at higher elevations.

## 2 Methods

### 2.1 Selection of Samples

Two of the seven species sequenced (Table S1) belong to lineage 1 of the core Brassicaceae: *Cardamine hirsuta* of lower elevations and *Cardamine resedifolia* of higher elevations. Their chromosome numbers correspond to the ancestral crucifer karyotype (ACK) *n* = 8 (Lysak et al. 2016). Only for *C. hirsuta* ploidy‐levels other than 2n were reported (besides 2n, also 4n and 8n) (Kiefer et al. 2014). Low‐elevation *Noccaea brachypetala* and high‐elevation *Noccaea rotundifolia* belong to lineage 2. For both species, a karyotype of *n* = 7 was described, corresponding to the ancestral proto‐Calepineae karyotype (PCK: *n* = 7) (Mandáková and Lysak 2008). Lower‐elevation *Arabis ciliata* and high‐elevation *Arabis caerulea* belong to the expanded lineage 2 (also referred to as lineage 4; Hendriks et al. 2023; Nikolov et al. 2019) and have a karyotype of *n* = 8 (Lauber et al. 2007; Titz 1972). Additionally, *Arabidopsis arenicola* with a distribution in subarctic and arctic Canada and Greenland was sequenced; it is a recent species that split from *A. lyrata* subsp. *lyrata* of mostly temperate North America at the end of the last Pleistocene glaciation (Willi et al. 2022). All species, with the exception of *N. rotundifolia*, were found to be autonomous selfers, on the basis of raising plants of two to six replicate populations per species in the greenhouse.

Analysis started with establishing a phylogenetic hypothesis on the 7 species, together with 35 further Brassicaceae species, for which a haploid genome assembly had been published (Table S2). For testing whether genome size and TE content were related to the geo‐climatic variables, we excluded six mesopolyploid species, as well as *Arabidopsis lyrata* , *Boechera divaricarpa* and *Microthlaspi erraticum* because of missing genome size estimation on the basis of *k*‐mer frequencies (Table S3). For the study of gene expansion/contraction, we used protein sequences of the 7 newly sequenced species and of 9 additional species for which protein sets were available on NCBI. These were: *Arabidopsis lyrata* (Kolesnikova et al. 2023), *Arabidopsis thaliana* (The Arabidopsis Genome Initiative 2000), *Arabis alpina* (Willing et al. 2015), *Brassica rapa* (Belser et al. 2018), *Capsella rubella* (Slotte et al. 2013), *C. hirsuta* (Gan et al. 2016), *Eutrema salsugineum* (Yang et al. 2013), *Schrenkiella parvula* (Dassanayake et al. 2011), and the outgroup *Aethionema arabicum* (Haudry et al. 2013) (Table S2). We included *B. rapa* only in the gene family contraction/expansion analysis to have a more balanced sampling of lineages, but excluded it from the genome size and TE analysis as it is a cultivar.

### 2.2 The Seven New Reference Genomes

#### 2.2.1 DNA and RNA Extraction and Sequencing

We extracted high molecular weight DNA using a CTAB‐based protocol according to Kleinboelting et al. (2012) and Siadjeu et al. (2020) with the following adjustments: RNAse was added after the 1‐h incubation with CTAB1. We incubated the samples after adding CTAB2 overnight, and we used 2 mL Eppendorf tubes instead of Falcon tubes for the extractions. RNA was extracted using the RNeasy Plant Mini Kit from Qiagen. For all species, we used leaves; for some species, we also used stem tissue ( *A. arenicola* , *A. caerulea* , *A. ciliata* , *C. resedifolia*), flowers ( *A. caerulea* , *C. resedifolia*, *N. rotundifolia*), and flower buds (*N. rotundifolia*). Both genome and transcriptome sequencing were done at the Functional Genomics Center Zurich (FGCZ) with the PacBio Sequel II to produce High‐Fidelity (HiFi) reads. Transcriptomic HiFi reads were de‐multiplexed, classified, and clustered into transcript sequences using the Iso‐Seq analysis app in the PacBio open‐source SMRT analysis software suite SMRT Link v10.1.0.119588 (Pacbio 2021).

#### 2.2.2 Genome Assembly

*K*‐mer (*K* = 17) frequency of genomic HiFi reads was calculated using kmerfreq v4.0 (Liu et al. 2013) (Table S4). Genome properties were estimated on the basis of calculated *k*‐mer frequency using gce‐alternative (Fan 2020). Genome assembly was performed using hifiasm v0.15.2 (Cheng et al. 2021) with the purge level set to 3 (−l 3). The primary sets of contigs were used to represent the reference genome sequences. Assembled reference sequences were quality‐controlled using QUAST v5.0 (Mikheenko et al. 2018) (Table S5) and BUSCO v5.0 (Manni et al. 2021) (Figure S1). Lineage datasets viridiplantae_odb10 and brassicales_odb10 (eukaryota, 2020‐09‐10) were used in the BUSCO analysis.

#### 2.2.3 Repeat Annotation

Repeat sequences per reference genome were identified using RepeatModeler v2.0.2a, with the new LTR discovery pipeline enabled (Flynn et al. 2020). Identified repeat sequences in the reference genomes were softmasked using RepeatMasker v4.1.2 (Smit et al. 2013) with the following parameters: ‐no_is ‐nolow ‐norna ‐excln. Repeat landscapes were analyzed using utility scripts within the same software package.

#### 2.2.4 Gene Prediction

Ab initio gene prediction was performed using AUGUSTUS v3.4.0 (Stanke et al. 2006) with species‐specific parameter sets. The high‐quality transcripts per species were aligned to the assembled reference sequences using minimap2 v2.21 (Li 2018). Initially, gene models were predicted using Iso‐Seq hints (extrinsicCfgFile = extrinsic.M.RM.PB.cfg) and the pre‐built *Arabidopsis* AUGUSTUS parameter set (Hoff and Stanke 2019). Then species‐specific parameters were computed. The aligned transcripts were converted to hints of gene structures using emtrey v1.1 (Volden 2019) and the utility script blat2hints.pl in AUGUSTUS. Genes with 100% support and with distinct amino acid sequences (< 80% identical to others on the amino acid level) were used as the training gene file, which was checked by inspecting the output files (i.e., checking for the error message “Terminal exon doesn't end in stop codon. Variable stopCodonExcludedFromCDS set right?”) from the first etraining analysis using the complete gene set and confirmed to be free of erroneous gene structures. Four hundred of the trusted genes were randomly selected as the test gene set; the remaining trusted genes were used to train the species‐specific parameters. The newly trained parameters were used to analyze the test gene set for measuring the sensitivity and specificity of AUGUSTUS species‐specific meta parameters, which were then optimized using the same training gene file per species with a maximum of five optimization rounds, with an 8‐fold cross‐validation for each new combination of parameters. Compared to the initial pre‐built Arabidopsis parameter set, the optimized species‐specific parameter sets improved both sensitivity and specificity of gene prediction results (Figure S2).

#### 2.2.5 Gene Annotation

For functional annotation, the ab initio predicted protein sequences were first compared against UniProtKB/Swiss‐Prot release‐2021‐04 (UniProt Consortium 2021) using NCBI BLASTP v2.10.1+ (Altschul et al. 1990; Camacho et al. 2009), with an *e*‐value cutoff of 10^−6^. UniProtKB/Swiss‐Prot protein ID and description of the best hit were assigned to each predicted protein sequence. Gene ontology (GO) annotation was then obtained by scanning PFAM domains (Blum et al. 2020) in each predicted protein sequence using interproscan v5.52‐86.0 (Jones et al. 2014). We also added GO annotations transferred from one‐to‐one orthologs in *A. thaliana* . The protein sequence file of *A. thaliana* (Araport11_pep_20250214.gz) was downloaded from: https://www.arabidopsis.org/download/list?dir=Proteins%2FAraport11_protein_lists.

The predicted protein sequences from our seven assembled genomes were combined with *A. thaliana* protein sequences into one protein database. Orthogroups among the eight species were identified using OrthoFinder v2.5.4 (summary statistics Table S6). GO annotation file of *A. thaliana* (ATH_GO_GOSLIM.txt.gz) was downloaded from: https://www.arabidopsis.org/download/list?dir=GO_and_PO_Annotations%2FGene_Ontology. GO terms annotated to *A. thaliana* proteins were then transferred to one‐to‐one orthologs in the other seven proteomes. Mapping of GO terms to functional categories (MF: molecular function, BP: biological process, and CC: cellular component) was obtained from GO.db v3.20.0 in R v4.4.2 (R Core Team 2024).

### 2.3 Comparative Genomics

#### 2.3.1 Geo‐Climatic Variables and Mating System

The analysis included the 7 newly sequenced genomes and 26 previously published genomes (Table S3). The medians of elevation, latitude, mean annual temperature (MAT), and mean annual precipitation (MAP) were calculated using the WorldClim database (Fick and Hijmans 2017) on the basis of the GBIF entries for each species (Table S7). GBIF entries were first filtered manually for the correct subspecies and the species' native distribution according to Plants of the World Online (2024). Then, sampling points were thinned to only keep entries which were at least 5 km away from each other using the function geoThin from the package enmSdmX (Smith 2022) in R v4.3.0. For *A. thaliana* and *C. hirsuta* , 10% of the data was randomly taken of all entries from GBIF before thinning, as it had too many entries for this step to run with reasonable memory efficiency (GBIF download links and Rscripts used for filtering are deposited on Dryad). Mean annual temperature was used as a first geo‐climatic variable as it is highly correlated with both elevation and latitude (Hopkins 1938; Montgomery 2007) as well as with other thermal variables, such as growing season length (Chu et al. 2016; Michaletz et al. 2014). The second geo‐variable was elevation corrected for MAT for each species, and the third variable in the main analysis was MAP corrected for MAT.

Outcrossing in Brassicaceae species is predominantly determined by sporophytic self‐incompatibility (SSI), which allows self‐pollen recognition (Fobis‐Loisy et al. 2004). It is hypothesized to be the ancestral state (Stebbins 1974), and several species have lost SI, turning themselves self‐compatible (e.g., Bachmann et al. 2019; Guo et al. 2009; Kolesnikova et al. 2023; Kusaba et al. 2001). We did a literature search to determine the mating system (self‐incompatible or self‐compatible) for each species (Table S2). If a species exhibited variation in its mating system, we used the predominant mating system of the population from which the genome assembly sample was obtained.

#### 2.3.2 Genome Size Estimation

We used the estimated genome sizes on the basis of *k*‐mer frequencies, either available from the literature or on the basis of our own estimations (Table S3). We used the short read archives (SRA) ERR5170146 for *A. arenosa* , SRR8083441 and SRR8083399 for *A. halleri* , and all available SRA for *C. rubella* , BioSample SAMN02981483. We ran Jellyfish v2.3.0 count and histo (Marçais and Kingsford 2011), and the output of the latter was used as input for GenomeScope 2 (Ranallo‐Benavidez et al. 2020). For *A. thaliana* , we compared the estimated genome sizes of 7 Col‐0 samples analyzed in Sun et al. (2018) with the estimated size published by EnsemblPlants (~135 Mb) and decided to use an estimated genome size of 135 Mb.

#### 2.3.3 Transposable Elements

Earl Grey v4.0.3 (Baril et al. 2024) was used to annotate transposable elements. Earl Grey is a pipeline that uses existing software for transposable element annotation and is highly user‐friendly and efficient. It combines de novo, similarity‐ and structure‐based approaches (Loreto et al. 2023).

#### 2.3.4 Phylogenetic Tree on BUSCO Amino Acid Sequences

BUSCO was run for each assembly using the brassicales lineage data set (brassicales_odb10). Only species with a complete BUSCO score above 70% were included ( *L. campestre* with 45% and *P. ovalifolia* with 33.7% were excluded). Three species missing per gene alignment were accepted. The amino acid sequences were aligned using MAFFT v7.490 (Katoh and Standley 2013) for each gene and concatenated using FASconCAT‐G v1.02 (Kück and Longo 2014). FastTree v2.1.11 (Price et al. 2009) was used to reconstruct the phylogeny. FastTree starts with a neighbor‐joining algorithm and calculates local support values similar to bootstrap values by resampling the data and testing how often the same branching appears. To make the tree ultrametric, a custom R script was used (code in supplement). ContMap of the package phytools (Revell 2024) was run to perform an ancestral state reconstruction for elevation and latitude with the same tree.

#### 2.3.5 Correlation Analysis

For a first impression on how characteristics of the genomes line up with each other and with all the geo‐climatic variables (without correction for MAT), a correlation analysis was performed. To correct for phylogenetic signal, phylogenetic independent contrasts (PIC) (Felsenstein 1985) were used, implemented in ape v5.8‐1 (Paradis and Schliep 2019) for the characteristics of the genomes and the geo‐climatic variables. This method transforms the trait data into independent contrasts, removing phylogenetic autocorrelation. The package corrplot (Wei and Simko 2021) was used in R, and elevation, the absolute term of latitude (|latitude|), MAT, MAP, genome size, and the fractions of all TEs and of each TE class were tested against each other.

#### 2.3.6 Phylogenetic Generalized Least Squares Models (PGLS)

First, a phylogenetic generalized least squares model (PGLS) using the package caper (Orme et al. 2023) in R with genome size as dependent and the mean‐centered fraction all TE(‐like) as independent variable was run. Second, the variables of genome size, fraction all TE, fraction LTR‐TE (class I, long‐terminal repeat retrotransposons; Kidwell 2005) and fraction DNA‐TE (class II, cut‐and‐paste DNA transposons; Kidwell 2005), treated as dependent variables, were linked with the (independent) variables of MAT, elevation corrected for MAT, MAP corrected for MAT, and mating system in various combinations (models in Table S8). Lambda (*λ*) was estimated using maximum likelihood. Models were compared by Akaike's information criterion weighted for small sample sizes (AICc). The ones with the smallest AICc were selected, and the output statistics were reported. For plotting, we used gls from the package nlme v3.1‐164 (Pinheiro et al. 2021) while taking the phylogenetic correlation into account using corPagel, allowing us to estimate confidence intervals. We colored the points according to clades (Table S3) to illustrate the phylogenetic structure. Further, once the best models were determined, we used Anova from the car package v3.1‐2 (Fox and Weisberg 2019) for type‐III testing to evaluate the unique contribution of each predictor while controlling for all other variables.

#### 2.3.7 Gene Family Expansion/Contraction

This analysis involved 16 species: the 7 newly sequenced ones (including *A. arenicola* ) and 9 previously published genomes (Table S2). Orthofinder v2.5.4 (Emms and Kelly 2019) and CAFE5 v5.1.0 (Mendes et al. 2021) were used to find expanded or contracted gene families. Orthofinder was set to use Diamond v0.9.29 (Buchfink et al. 2015), MAFFT v7.453, and IQtree v2.1.2 (Minh et al. 2020). Further, we used Cafeplotter v0.2.0 (https://github.com/moshi4/CafePlotter, accessed on 1 January 2024) to create graphs.

Zooming in on the expansion or contraction of gene families associated with elevation, we focused on the three species pairs with new reference genomes: *A. caerulea* (high elevation) compared to *A. ciliata* (low elevation), *C. resedifolia* to *C. hirsuta* , and *N. rotundifolia* to *N. brachypetala*. Gene families with signatures of expansion or contraction in the high‐elevation as compared to low‐elevation species of the pairs were linked with their GO terms. The GO terms of all gene families found in the three comparisons were identified using https://www.arabidopsis.org/tools/go_term_enrichment.jsp powered by PANTHER v19.0 (Thomas et al. 2022) (analysis type: PANTHER Overrepresentation Test released on 2024‐02‐26; annotation version: GO Ontology database DOI: https://doi.org/10.5281/zenodo.10536401 released on 2024‐01‐17; reference list: *A. thaliana* (all genes in database); test type: Fisher's exact; correction: Bonferroni). We traced back which GO terms were associated with expanded and contracted gene families in more than one of the species pairs (high and low elevation) and highlighted them in the plot with an asterisk.

#### 2.3.8 GO‐Figure

The Python package GO‐Figure (Reijnders and Waterhouse 2021) was used to illustrate the GO terms and their clustering. The program summarizes a list of GO terms in a scatterplot, showing functionally related GO terms closer to each other in semantic space. The protocol was followed as explained on https://gitlab.com/evogenlab/GO‐Figure.

## 3 Results

### 3.1 New Reference Genomes

Summary statistics on the new reference genomes are presented in Table S5. Fifty percent of the assembled genomes were in 7–12 contigs, indicating near‐chromosome‐level assemblies, given that *A. arenicola* , *A. caerulea, A. ciliata*, *C. hirsuta*, and *C. resedifolia* have 8 and *N. brachypetala* and *N. rotundifolia* have 7 chromosomes. Estimated genome sizes on the basis of *k*‐mers were between 0% and 17% higher than assembly sizes, except for *N. rotundifolia*, for which the estimated genome size was 6% smaller than the assembly size. Single‐copy‐ortholog scores varied between 91.3% and 98.5%. Finally, 40.5% to 63.5% of the bases were masked, indicating the fraction of repetitive elements in the genomes.

### 3.2 Comparative Genomics

#### 3.2.1 Phylogenetic Tree on BUSCO Amino Acid Sequences

**FIGURE 1:**
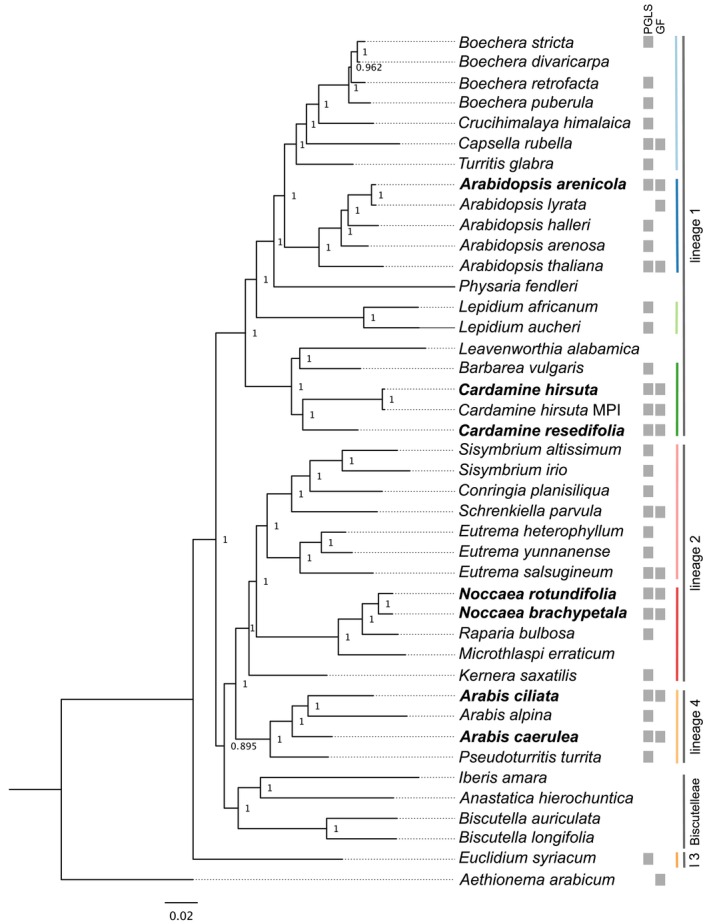
FastTree phylogeny on the basis of 665 BUSCO genes (lineage data set brassicales_odb10). Numbers at nodes indicate local support (0–1) (Price et al. 2009). Genomes assembled in this study are written in bold. Gray boxes on the right show in which analysis they were included [PGLS models and gene family expansions and contractions (GF)]. Coloured lines show clades used for indicating phylogenetic relationships in Figure 3.

Using the BUSCO genes of the newly sequenced assemblies and the protein sets for the added 35 Brassicaceae species, we were able to reconstruct a phylogeny similar to already published phylogenies (Figure 1) (Hendriks et al. 2023; Walden et al. 2020). Next, we focused on the link between genomic features and geo‐climatic variables. The ancestral state reconstruction using 40 species and their median latitude as well as median elevation revealed that both high elevation and high latitude were generally derived colonized environments, and the Brassicaceae's origin was at low elevation and at medium latitude (Figure S3).

#### 3.2.2 Correlation Analysis

**FIGURE 2:**
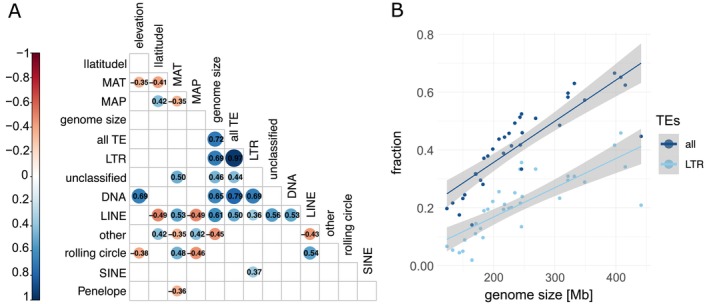
(A) Pairwise correlations among environmental variables (|latitude| = absolute value of latitude, MAP = mean annual precipitation, MAT = mean annual temperature) and properties of genomes, with a focus on types of repetitive elements, first the fraction of all TEs, then the fraction of the transposon classes of (in order of declining abundance) LTR, unclassified, DNA, LINE, other (simple repeat, microsatellite, RNA), rolling circle, SINE, and Penelope. Red circles indicate significant negative correlations and blue circles positive correlations; numbers are Pearson correlation coefficients. (B) The fraction of all TEs and of the class LTR in relation to genome size.

For an overview of if and how characteristics of the genome and medians of elevation, latitude, mean annual temperature (MAT), and mean annual precipitation (MAP)—all corrected for phylogenetic relations—line up with each other, we did a correlation analysis including the seven newly sequenced species and the 26 species from Table S3 (ranges of latitude and elevation can be found in Table S7). Elevation was correlated positively with the fraction of DNA TEs and negatively with the fraction of rolling circle elements (Figure 2A). Latitude was correlated positively with the fraction of other TEs and negatively with the fraction of LINE‐TEs. In turn, MAT, which had negative links with elevation, latitude, and MAP, was correlated positively with the fractions of unclassified TEs, LINE elements, and rolling circle elements and negatively with the fractions of other TEs and Penelope. Finally, MAP shared the same correlations with genomic variables as latitude, but with the additional negative correlation with the fraction of rolling circle elements. Within the genomic variables, correlations were—if significant—15 times positive and twice negative, with the strongest correlations between the fractions of all TEs and LTR‐TEs (*r* = 0.97) and the fractions of all TEs and DNA‐TEs (*r* = 0.79, Figure 2A). Further significant positive correlations were found between genome size and the most abundant TE groupings: fraction all TE, LTR, unclassified, DNA, and LINE (Figure 2A). Or in other words, large genome size was mainly the result of more TEs (Figure 2B).

#### 3.2.3 Phylogenetic Generalized Least Squares Models on Geo‐Climatic Variables and Mating System

**FIGURE 3:**
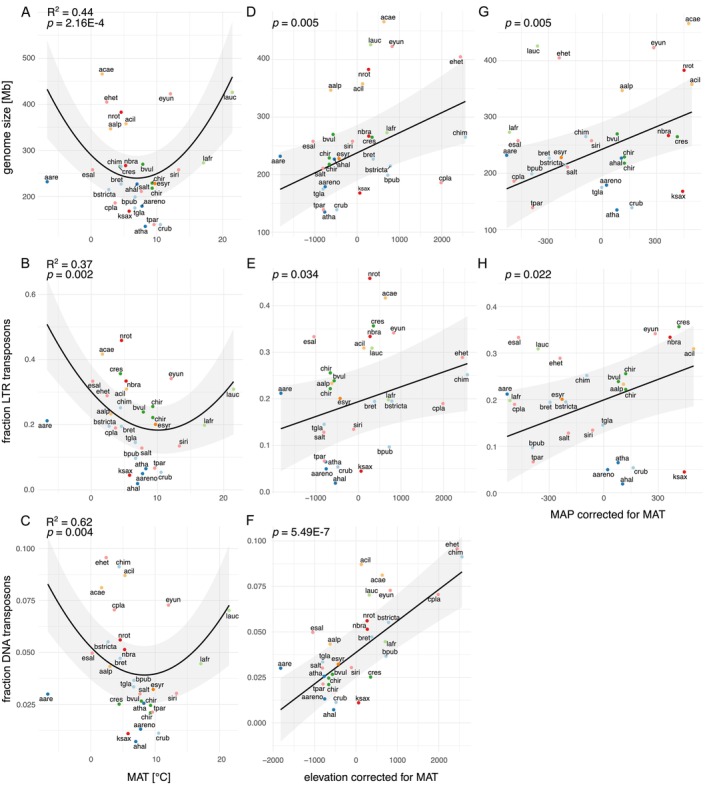
Genomic variables in relation to (medians of) mean annual temperature (MAT) of species' occurrences (on the left), elevation (corrected for MAT; in the centre), and mean annual precipitation (MAP corrected for MAT; on the right). Black lines represent significant quadratic or linear predictions according to the best model, and the gray areas indicate 95% confidence intervals (Table 1). R^2^ values refer to the variance explained by the best model. The species (coloured circles) were grouped into clades to represent the phylogenetic autocorrelation (see Figure 1, Table S3).

Model comparisons were built by increasing complexity, and the best model for genome size and most repetitive elements included the linear and quadratic terms of MAT and the linear terms of elevation and MAP, both corrected for MAT (Table S8). Therefore, we refer here to the results of that model (Table 1). This model revealed that for the tested genomic variables, the quadratic term of MAT was always positive and significant. Genomes were smallest in species occurring in climates of moderate mean annual temperatures (Figure 3A). Similarly, genomes of species with a distribution in moderate climates had the smallest fractions of all TEs, LTR‐TEs, and DNA‐TEs (Figure 3B,C). For elevation, we found a significant positive effect on genome size and the fractions all TE, LTR‐TE, and DNA‐TE (Figure 3D–F). The best model for the fraction DNA‐TE in relation to the environmental variables did not include MAP. This model, however, explained the highest amount of variation (62%), and the relationship of the fraction DNA‐TE with elevation was the most significant. Mean annual precipitation had a significant positive effect on genome size and the fractions all TE and LTR‐TE (Figure 3G,H), indicating that species from wetter habitats have larger genomes and higher TE contents. Type‐III testing confirmed that the contribution of each of these environmental variables while controlling for all others was significant (Table 1).

**TABLE 1:** Results of best‐supported PGLS models (on the basis of AICc).

*β*		Genome size (Mb)	Fraction TE	Fraction LTR‐TE	Fraction DNA‐TE
Intercept	*β*	240.48***	0.38***	0.19***	0.04***
	SE	23.77	0.04	0.032	0.0056
	*χ* ^2^	95.36	71.60	29.45	30.12
MAT	*β*	0.25	−0.0058	−0.0066*	−6.85E‐04
	SE	2.31	0.0039	0.0031	5.39E‐04
	*χ* ^2^	0.02	2.40	4.89*	0.82
MAT^2^	*β*	1.04***	0.0018***	0.0011**	1.65E‐04**
	SE	0.24	4.08E‐04	3.26E‐04	5.22E‐05
	*χ* ^2^	18.93***	23.00***	14.27***	16.90***
Res_Elevation	*β*	0.035**	5.60E‐05**	3.42E‐05*	1.77E‐05***
	SE	0.01	1.91E‐05	1.53E‐05	2.69E‐06
	*χ* ^2^	9.45**	7.57**	4.38*	38.43***
Res_MAP	*β*	0.13**	1.63E‐04*	1.44E‐04*	
	SE	0.04	7.36E‐05	5.87E‐05	
	*χ* ^2^	9.15**	4.86*	6.10*	
Adjusted *R* ^2^		0.44	0.45	0.37	0.62

*Note:* The models tested for relationships between characteristics of genomes (dependent variables) and (mean‐centered) environmental variables (independent variables). *β* is the estimate, SE the standard error, *χ* ^2^ the result of the analysis of deviance (type‐III‐tests). Estimates in bold are significant [(*) *p* < 0.1, *p < 0.05, **p < 0.01, ***p < 0.001].

#### 3.2.4 Similar GO‐Terms in Expanded and Contracted Gene Families in High‐Elevation Species

**FIGURE 4:**
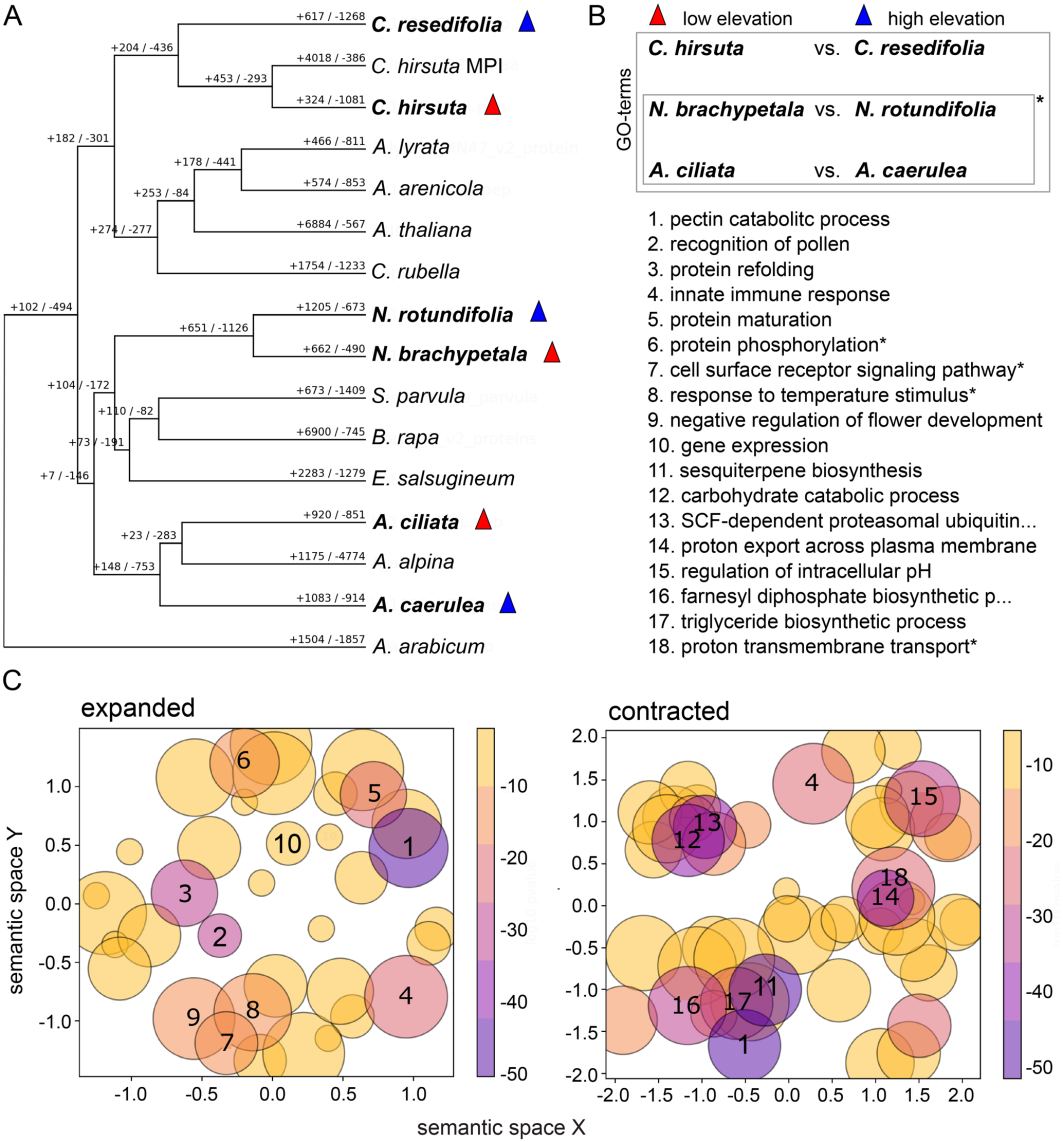
Summary of gene family expansions and contractions on the basis of the protein‐annotated reference genomes available on NCBI and gene ontology terms of expanded and contracted gene families. (A) Number of gene families expanded (+) and contracted (−) for each branch. (B) For the semantic grouping, we included gene families that were expanded or contracted in at least one of the three high‐elevation species (blue triangles), but either did not change or were changed in the opposite way in the related low‐elevation species (red triangles). GO‐terms with a star were enriched in expanded (6, 7, 8) or contracted (18) gene families in the comparisons between *Arabis caerulea* to *Arabis ciliata* and *Noccaea rotundifolia* to *Noccaea brachypetala*. (C) The semantic space clustering of GO‐terms was done using all expanded and contracted gene families of the three comparisons. Coloration of circles indicates enrichment *p*‐value (log_10_), with the scale on the right of each panel. *p*‐values were calculated using Fisher's exact test, with Bonferroni correction for multiple testing. Semantic space X and Y group GO‐terms by semantic similarity, allowing to group by biological function. The size of circles indicates the number of GO‐terms related to the representative GO‐term. The circles with numbers indicate the 10 GO‐terms with the smallest *p*‐value. We display only meaningful GO‐terms (specific enough to be useful for indicating the function of genes).

The number of expanded and contracted gene families of the 16 species used for this analysis is shown in Figure 4A. We found that 277 gene families had experienced expansions in the three comparisons of high‐ to low‐elevation species with newly sequenced reference genomes. Here, we focus on the top gene‐ontology terms, for which semantic clustering is presented in Figure 4C (also see Tables S9 and S10). A first GO‐term was “pectin catabolic processes” (Figure 4B). Pectin is common in the cell wall and the primary compound of the middle lamella (Caffall and Mohnen 2009; Parker et al. 2001). Pectin is important for the specificity and plasticity of cell wall remodeling (Cosgrove 2016; Wolf and Greiner 2012), and as such, it acts as a major contributor to the process of intercellular communication and environmental sensing (Shin et al. 2021). Interestingly, gene contractions were observed for the same process, suggesting the remodeling of the catalysis of pectin in high‐elevation species. Four other significant ontology terms of expanded genes were related to proteins, their refolding, maturation and phosphorylation, and gene expression. Biologically more explicit terms capturing gene expansions in the high‐elevation species were “pollen recognition”, “innate immune response”, “response to temperature stimulus”, and, lastly, “negative regulation of flower development”. “Innate immune response” also popped up in contracted genes, suggesting the remodeling of immunity in high‐elevation species. “Negative regulation of flower development” may indicate suppression of early flowering and be linked to a more perennial lifestyle in alpine plants (Körner 2021).

We found that 211 gene families had experienced contractions in high‐ as compared to low‐elevation species. As mentioned, two GO terms with contracted genes overlapped with expanded genes, “pectin catabolic processes” and “innate immune response”. Further, contracted gene families were captured by the GO terms of “sesquiterpene biosynthetic process” in the top position, and the semantically related terms “farnesyl diphosphate biosynthetic process” and “triglyceride biosynthetic process”. Sesquiterpene can help plants under oxidative as well as microbial stress (Ahuja et al. 2012; Schmelz et al. 2014), farnesyl diphosphate helps regulate growth and development (Banyai et al. 2010; Closa et al. 2010), whereas triglycerides are an important component of oils and involved in storage in the seeds (Li‐Beisson et al. 2013; Zhu et al. 2020). Another GO term associated with a contracted gene family was “SCF‐dependent proteasomal ubiquitin‐dependent protein catabolic process”. Genes associated with this GO term are involved in cell cycle progression, DNA damage response, signal transduction, and transcription (Xie et al. 2019). The following three GO terms were related to pH homeostasis, a precondition for normal growth and stress response (Zhou et al. 2021): “proton export across plasma membrane”, “regulation of intracellular pH”, and “proton transmembrane transport”. Finally, “carbohydrate catabolic process” is related to plant defense response (Rojas et al. 2014).

## 4 Discussion

Genome size in angiosperms has been shown to span four orders of magnitude, from ~60 to ~125,000 Mb (Gregory et al. 2007; Michael 2014). Furthermore, large genome size has been associated with an increase in the amount of repetitive elements (Bennett and Leitch 2005; Tenaillon et al. 2010), especially of the repeat class LTR (long terminal repeat retrotransposons) (Michael 2014). We confirmed this finding in the family Brassicaceae, as we observed an increase in TEs proportional to genome size, mainly because of an increase in LTR‐TEs (Figure 2B) and smaller contributions of unclassified TEs, DNA elements, and LINE elements. Furthermore, we studied how genome size and the fraction of TEs are related to the geo‐climatic distribution of species. We found that species from warm and cool climates, as well as those of higher elevations and places with more precipitation—beyond the effect of average temperature—had more TEs and larger genomes (Figure 3). Additionally, we investigated what gene families had expanded or contracted in high‐ compared to low‐elevation plant species and which GO terms were enriched. Below, we discuss results in the context of genome evolution associated with biogeography, with a special focus on elevation.

### 4.1 Genome Size of Species in Extreme Environments

Small genome size is typically the ancestral state, as larger genome sizes were found to be more abundant in more derived taxa within larger clades (Soltis et al. 2003). However, evolution from larger to smaller genome sizes has also been reported (Michael 2014), suggesting that genome size is an evolutionarily labile trait. The drivers of the evolution of genome size are still largely unknown (Pellicer et al. 2018). Our study clearly contributes here by emphasizing that genome size is influenced by geo‐climatic distribution, and this mainly via the evolution of the abundance of TEs that contribute most to size differences.

First of all, we found that Brassicaceae species evolved from low elevations and medium latitude toward high elevation and high latitude (in absolute terms) (Figure S3). This is in line with a general pattern in plants: The biome‐shift between low‐land and alpine species seems to be asymmetrical, with low‐land species more likely to adapt to high elevations than the other way around (Bätscher and de Vos 2024). The species at the most extreme temperatures in this study were *A. arenicola* and *L. aucheri* . *Arabidopsis arenicola* is the species with the most northern distribution in this study and occurs at the coldest temperatures (Table S7, Figure 3A–C). It is a young species that split from North American *A. lyrata* subsp. *lyrata* about 6000 generations ago and has expanded its range beyond the latitudes where the parental species occurs (Willi et al. 2022). At the warm end is *Lepidium aucheri* , a desert and dry shrubland inhabitant, with a distribution from Egypt to Kazakhstan (Plants of the World Online 2024). The species occurring at the highest elevations are *C. himalaica*, *E. heterophyllum*, and *C. planisiliqua*, all occurring in Central Asia, including the Himalaya mountains. As small genome size is assumed to be the ancestral state (Soltis et al. 2003), we might conclude that Brassicaceae evolved towards more extreme environments while simultaneously increasing genome sizes in different lineages.

Some years ago, a different idea was posited, named the “large genome constraint hypothesis” (Knight et al. 2005). It states that species with small genomes may occur at any elevation or latitude, but species with large genomes might be excluded from the extremes. In line, in a study on northwestern Patagonian *Berberis*, species at high elevations, with greater rainfall but less water availability because of frozen soils in winter, had indeed smaller DNA content (Bottini et al. 2000). Hence, the pattern may possibly be related to water availability and not elevation per se. A precipitation gradient experiment showed that species with smaller genomes use water more efficiently than those with larger genomes, making them more drought‐tolerant—a trait primarily attributed to their smaller cell sizes (Sun et al. 2025). In contrast to findings in *Berberis* by Bottini and coworkers, genome size in the Brassicaceae genus *Streptanthus* and close relatives increased with greater temperature seasonality in the California Floristic Province, meaning that large genomes are associated with habitats of sub‐zero winter and spring temperatures (Cacho et al. 2021). Also, in a study of 14 *Hypochaeris* species, an increase of genome size with elevation was observed (Cerbah et al. 1999). In our study, species of higher elevations—other than for temperature—did have larger genomes, together with those of hotter and cooler as well as wetter climates, contributing to a coherent picture emerging on the geo‐climatic drivers of genome size.

### 4.2 More TEs—Adaptive Potential or Byproduct

Large genome size has, for example, been linked to cellular and nuclear sizes (Cavalier‐Smith 1982; Gregory and Hebert 1999), duration of mitosis and meiosis (Bennett 1987), and generation time (Bennett and Riley 1997; Wakamiya et al. 1993). Here, we could show—together with a number of previous studies (Kidwell 2002; Lee and Kim 2014; Sun et al. 2023)—that variation in genome size was positively linked with the fraction of all TEs and its most prevalent class in plants, LTR‐TEs. Genome size, the fraction of all TEs, and the fraction of LTR‐TEs also showed concordant patterns with climate. Values were higher in species of cooler and warmer than average climates, and they increased with elevation, decoupled from temperature. This strongly speaks in favor of the idea that the increase in genome size with more extreme climate and higher elevation is due to TEs. An interesting added factor was that DNA‐TEs were particularly abundant in high‐elevation species, with elevation (corrected for temperature) explaining variation in this TE class very well (Figure 3H). DNA‐TEs move via a “cut‐and‐paste” mechanism and reinsert themselves in a different place (Wicker et al. 2007). It has been shown that they are especially abundant in gene‐rich regions of plant genomes (Feschotte et al. 2002), where they likely directly influence gene function and expression (Jiang 2013). Their copy number is increased, for example, by replicative repair or through the duplication of genomic segments containing these elements (Slotkin and Martienssen 2007). An interesting example of potentially facilitated evolution by DNA‐TEs has been observed in the microbat genus *Myotis* (Oliver et al. 2013). Although DNA‐TEs were believed to have been inactive in mammals for the past 37 million years (Pace and Feschotte 2007), they underwent massive amplification in *Myotis* (Pritham and Feschotte 2007; Ray et al. 2007, 2008). This spread of TEs may have contributed to the evolutionary radiation of the genus by promoting dynamic genomes.

Also, the “copy‐paste” transposon class LTR has been shown to respond to different kinds of stress and might aid genome diversification in plants, animals, and fungi (Maumus et al. 2009). In wild barley (*Hordeum spontaneum*), genome size and the number of copies of retrotransposons of the family *BARE*‐1 were correlated with microclimate (Kalendar et al. 2000). The number of TEs and genome size increased with higher positions and simultaneously drier conditions on the slope in the Evolution Canyon in Israel. There were ABA (abscisic acid)‐response elements present within the *BARE*‐1 promoter, a typical sign for water stress‐induced genes. Therefore, the authors suggest adaptive evolution for a higher number of *BARE‐*1 TEs at the driest point, on the top of the slope (Kalendar et al. 2000; Suoniemi et al. 1996). A more indirect selective advantage of larger genomes with more TEs could be related to growth. Fast‐growing plants have generally small genomes and fewer TEs, whereas plants with larger genomes have longer minimum generation times and slower growth rates because of slower cell division (Francis et al. 2008; Šímová and Herben 2011). Alpine plants are usually shorter (Körner et al. 1989; Pellissier et al. 2010) and slow‐growing perennials (Billings 1974; Atkin et al. 1996, but see Maccagni and Willi 2022), either because of divergent adaptation or plastic responses. Because cell division rates are decreased by low temperatures, growth by means of an increase in cell volume may be favored and achieved by higher DNA content (Francis and Barlow 1988; Grime and Mowforth 1982). Indeed, there is a trend of more polyploid species towards both poles in angiosperms (Bureš et al. 2024). In the study by Bureš et al., genome size followed the trend of increase towards the northern pole, but it reached a threshold when genome size began to shrink again. Maybe, we did not cover this threshold with our sampling, or this trend does not hold true for the Brassicaceae family.

TE accumulation might also be explained by other factors not involving adaptive evolution. TE abundance might vary with the mating system. Although the spread of TEs might be inhibited by self‐fertilization because of the lack of interchange between individuals, TEs can accumulate more easily in selfers if selection against TEs is predominantly mediated by ectopic recombination (Bonchev and Willi 2018; Charlesworth and Charlesworth 1995). Another reason for the spread of TEs in genomes of species in extreme habitats can be a disturbance in the “epigenomic surveillance system” (Michael 2014). Many plant species with small genomes have been shown to effectively get rid of an overload of TEs and have shrunk their genome size again, like *A. thaliana* (Michael 2014), by DNA methylation to silence TEs and slow down their self‐proliferation (Lippman et al. 2004; Miura et al. 2001; Zilberman et al. 2007). Environmental stress has been suggested to lead to the liberation and activation of TEs (Slotkin and Martienssen 2007). For example, in *Drosophila simulans* , temperature influenced the rate of transposition in a lab experiment, which was confirmed in the wild, where the copy number follows a minimum temperature cline (Vieira et al. 1998). A related reason may be that species of extreme climates are younger and have not had enough time to purify their genomes from an overload of TEs. Further, TEs are more easily fixed by drift in populations that underwent a bottleneck (Matzke et al. 2012), and there is a hypothesis of a negative correlation between effective population size and genome size (Lynch and Conery 2003). However, a study on seed plants did not find support: A previously significant negative relationship between effective population size and genome size became non‐significant after accounting for phylogenetic relationships (Whitney et al. 2010).

### 4.3 Adaptation to High‐Elevation Environments in Brassicaceae

Gene family expansion and contraction can both be adaptive. For gene family expansion, maybe more copies increase dosage and have a positive effect on the selected phenotype. Gene family contraction could lead to reduced expression or members of gene families being lost because they are simply not needed anymore (Demuth and Hahn 2009). However, a “less is more” hypothesis (Olson 1999) has been proposed as well, in which case the number of gene losses was attributed to the fact that the null allele conferred a selective benefit (e.g., Stedman et al. 2004; Varki 2001; Wang et al. 2006). In the comparison of high‐ to low‐elevation species, we observed expansion as well as contraction in gene families associated with GO‐terms related to the breakdown of pectin which is involved in cell wall remodeling and to innate immune response (Figure 4). The latter might be caused by different diseases at higher elevations, which leads to an adapted immune system. Temperature can influence the potency of pathogens (Wang et al. 2009); hence, the on‐average low temperatures at high elevations combined with peak‐heat temperatures during summer might have triggered adaptations of the immune system. Exclusively expanded gene families were involved in processes related to posttranslational protein modification, from gene expression to protein maturation. As protein synthesis can be affected by temperature (Craig 1975; Farewell and Neidhardt 1998) and proteome alterations are key elements in cold adaptation (Barrero‐Gil and Salinas 2013; Janmohammadi et al. 2015), this expansion is likely an adaptation to a cooler environment.

Contractions in high‐elevation species were mainly associated with the biosynthesis of either protective agents or storage: for example, sesquiterpene lactones are terpenoid compounds that are toxic to insects, anti‐fungal, anti‐bacterial, and regulate plant growth (Picman 1986). Colder temperatures have negative effects both on insect development and the nutrient content of the leaves, maybe leading to a decreased risk of herbivory at higher elevations (Garibaldi et al. 2011). It has been shown in *A. alpina* that herbivore damage actually decreased with elevation (Buckley et al. 2019). Therefore, this might indicate a less prevalent threat from herbivory and diseases at high elevations, and plants need less protection.

## 5 Conclusion

In this study, we confirm that the extant Brassicaceae species have variable genome sizes because of variation in genome‐wide TE content. Furthermore, both genome size and TE content could be explained to similar extents by the geography of species' distribution. Species of cooler and warmer than average climates, as well as species of higher elevations independent of temperature and from regions with higher precipitation had larger genomes and more TEs. So far, it cannot be determined if the increase in TEs is adaptive or if the TE‐silencing machinery might be impacted by more extreme climatic factors. Regarding gene family expansions and contractions in high‐elevation species, we show several changes related to GO terms connected to cell wall remodeling, innate immune system, and protein synthesis. They could be associated with new abiotic conditions at high elevations, like colder temperatures, but also with a change in biotic interactions, for example, with herbivores or pathogens.

## Author Contributions

**Jana M. Flury:** conceptualization (lead), formal analysis (lead), investigation (lead), methodology (lead), writing – original draft (equal), writing – review and editing (equal). **Weihong Qi:** formal analysis (supporting), methodology (supporting), writing – original draft (supporting), writing – review and editing (supporting). **Olivier Bachmann:** methodology (supporting), writing – original draft (supporting), writing – review and editing (supporting). **Yvonne Willi:** conceptualization (equal), funding acquisition (lead), investigation (supporting), methodology (supporting), project administration (equal), resources (lead), writing – original draft (equal), writing – review and editing (equal).

## Conflicts of Interest

The authors declare no conflicts of interest.

## Supporting information


**Data S1:** ece372462‐sup‐0001‐DataS1.docx.

## Data Availability

Raw sequencing data, as well as genome assemblies and annotation, are available on ENA under bioproject accession number PRJEB79954. The download links for the data from GBIF as well as the Rscripts to filter them are available on Dryad https://doi.org/10.5061/dryad.79cnp5j8z.
