# Supplementary material for: Evolution of Repetitive Genomic Content and Gene Families Over Geo‐Climatic Gradients in Brassicaceae
Source: Ecol Evol. 2025 Dec 9;15(12):e72462. doi: 10.1002/ece3.72462 (PMC12689959; doi:10.1002/ece3.72462)
Supplement: Supplementary file 1 — Data S1: ece372462‐sup‐0001‐DataS1.docx. [file ECE3-15-e72462-s001.docx]

Supplement

Evolution of repetitive genomic content and gene families over geo-climatic gradients in Brassicaceae

Jana M. Flury, Weihong Qi, Olivier Bachmann, Yvonne Willi

**Content**

**Figures, pages 2-4**

Fig. S1: BUSCO results reference genomes

Fig. S2: Augustus - optimized species-specific parameter settings

Fig. S3: Ancestral state reconstruction for elevation and latitude with 40 Brassicaceae species

**Tables, pages 5-24**

Tab. S1: Sampling locations of individuals used for reference genome

Tab. S2: Overview samples

Tab. S3: Estimated genome sizes and clades

Tab. S4: Reference genomes – results of k-mer analysis

Tab. S5: Reference genomes – QUAST results

Tab. S6: Summary statistics Orthograph

Tab. S7: Median and interquartiles of latitude, elevation, mean annual temperature, and precipitation

Tab. S8: Model selection

Tab. S9: GO-terms associated with gene family expansion in high-elevation species

Tab. S10: GO-terms associated with gene family contraction in high-elevation species

**Code for bioinformatic analyses, pages 25-32**

**References, pages 33-35**


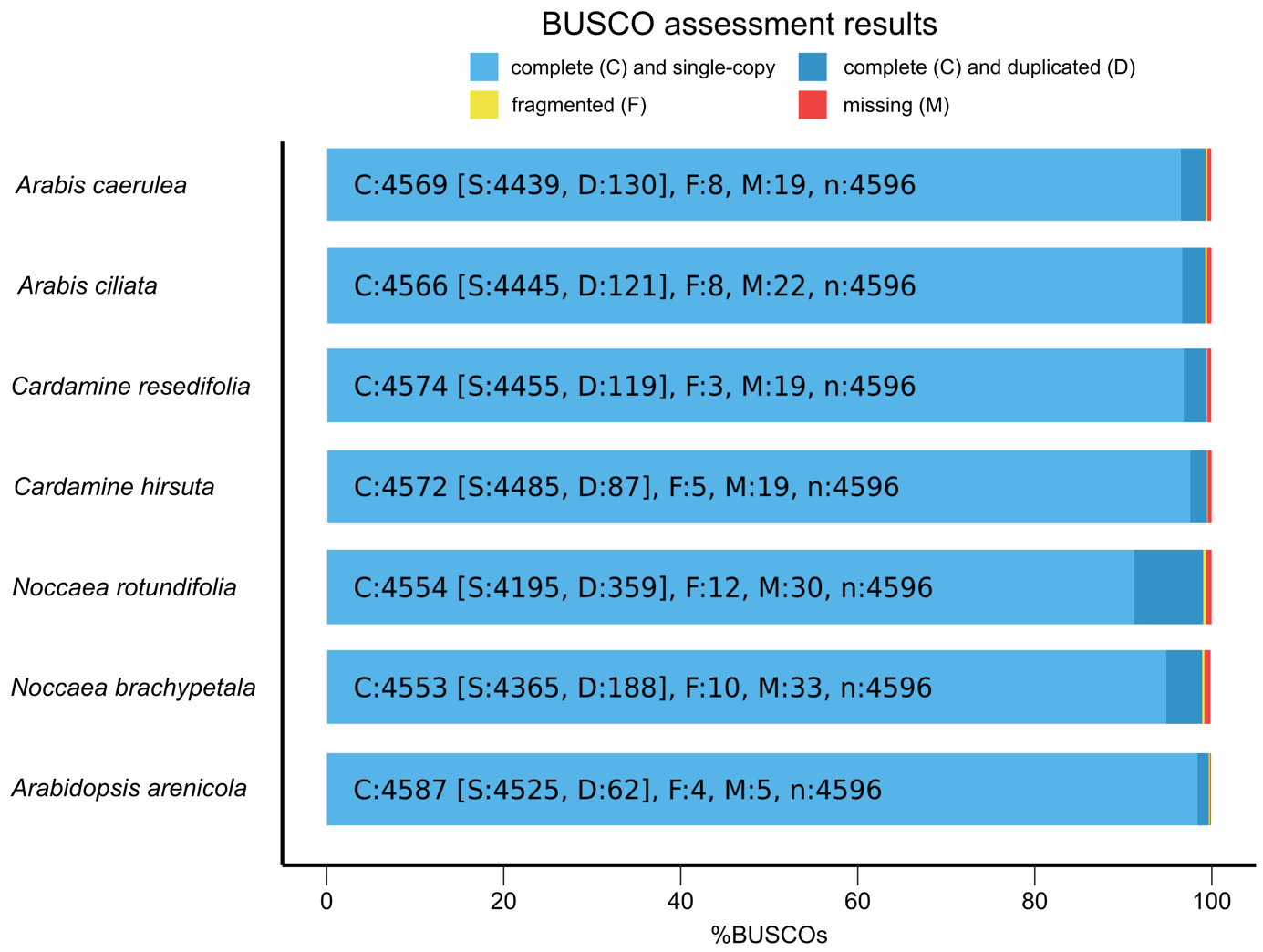
**Figures**

Fig. S1: The BUSCO results show the high quality of all seven new reference genomes. Most BUSCO genes are complete and single-copy.

Fig. S2: Optimized species-specific parameter settings improved both sensitivity and specificity of gene prediction results.


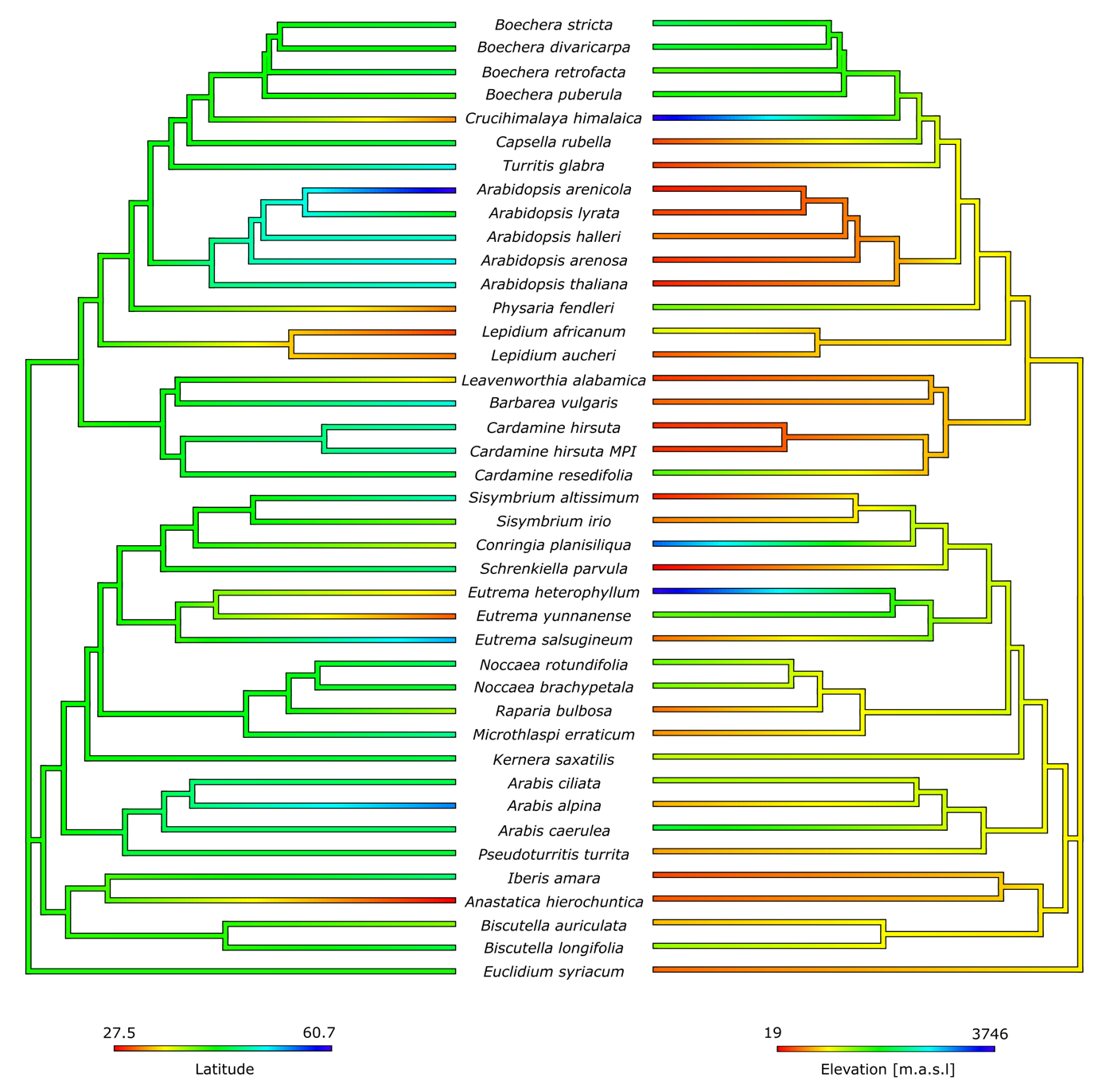


Fig. S3: Ancestral state reconstruction based on a FastTree phylogeny, with the geo-climatic variables of latitude on the left and elevation on the right.

Tables

Tab. S1: Samples used for the new reference genomes and sampling locations. Mating system SC means self-compatible, SI means self-incompatible.

| Sample | Species | Latitude  [° N] | Longitude  [° E/W] | Elevation  m a.s.l. | Country | Collection date | Mating system |
| --- | --- | --- | --- | --- | --- | --- | --- |
| bc1009 | *Arabis caerulea* | 46.761 | 10.343 | 2670 | CH | 19.09.16 | SC |
| bc1008 | *Arabis ciliata* | 46.014 | 7.795 | 2530 | CH | 27.08.16 | SC |
| bc1020 | *Cardamine resedifolia* | 46.833 | 9.804 | 2645 | CH | 22.09.19 | SC |
| bc1021 | *Cardamine hirsuta* | 47.178 | 8.493 | 415 | CH | 05.05.19 | SC |
| bc1010 | *Noccaea rotundifolia* | 47.243 | 9.360 | 2005 | CH | 24.08.16 | SI |
| bc1011 | *Noccaea brachypetala* | 46.921 | 7.923 | 1060 | CH | 20.06.16 | SC |
| bc1012 | *Arabidopsis arenicola* | 58.780 | -94.195 | 0 | CAN | 27.07.17 | SC |

Tab. S2: Information on the additional species included in the study: assembly name, mating system, and GenBank assembly accession. The last column indicates the analyses the data was used in: phylogenetic tree (1), PGLS models (2), gene family expansion and extraction (3).

|  | Species | Assembly name | Mating system | GenBank assembly accession | Used in |
| --- | --- | --- | --- | --- | --- |
| 1 | *Aethionema arabicum* | VEGI_AA_v_1.0 | SC^1^ | GCA_000411095.1^21^ | 1, 3 |
| 2 | *Anastatica hierochuntica* | ASM90040627v1 | SC^2^ | GCA_900406275.1^22^ | 1 |
| 3 | *Arabidopsis arenosa* | AARE701a | SI^3^ | GCA_905216605.1^23^ | 1, 2 |
| 4 | *Arabidopsis halleri* | Ahalleri_CH_v2 | SI^1^ | GCA_003711535.1^24^ | 1, 2 |
| 5 | *Arabis alpina* | MPIPZ.v5 | SC^4^ | GCA_900128785.1 | 1, 2, 3 |
| 6 | *Arabidopsis lyrata* | MN47_v2 | SI^35^ | GCA_944990045.1 | 1, 3 |
| 7 | *Arabidopsis thaliana* | TAIR10.1 | SC^1^ | GCA_000001735.2^25^ | 1, 2, 3 |
| 8 | *Barbarea vulgaris* | ddBarVulg1.1 | SI^6^ | GCA_963667165.1^26^ | 1, 2 |
| 9 | *Biscutella auriculata* | ASM90040628v1 | SC^7^ | GCA_900406285.1^22^ | 1 |
| 10 | *Biscutella laevigata subsp. laevigata* | Biscutella_longifolia | SI^7^ | GCA_900406315.1^22^ | 1 |
| 11 | *Boechera divaricarpa* | BoeM4B | SC^8^ | GCA_026770425.1 | 1 |
| 12 | *Boechera puberula* | ASM90040633v1 | SC^8^ | GCA_900406335.1^22^ | 1, 2 |
| 13 | *Boechera retrofracta* | SPBU_Bret_1.0 | SC^8^ | GCA_015832515.1^27^ | 1, 2 |
| 14 | *Boechera stricta* | NTU_Bstr_SAD12_2.2 | SC^8^ | GCA_018361395.1 | 1, 2 |
| 15 | *Brassica rapa* | brapa_z1_v2 | SI | GCA_900412535.3 | 3 |
| 16 | *Capsella rubella* | Caprub1_0 | SC^1^ | GCA_000375325.1^28^ | 1, 2, 3 |
| 17 | *Cardamine hirsuta* | chi_v1 | SC | See below | 1, 2, 3 |
| 18 | *Conringia planisiliqua* | Conringia_planisiliqua.v1 | SC^9^ | GCA_900108845.1^22^ | 1, 2 |
| 19 | *Crucihimalaya himalaica* | ASM434971v1 | SC^10^ | GCA_004349715.1^29^ | 1, 2 |
| 20 | *Euclidium syriacum* | Euclidium_syriacum.MPIPZ.v1 | SC^11^ | GCA_900116095.1^22^ | 2 |
| 21 | *Eutrema heterophyllum* | ASM293391v1 | SC^12^ | GCA_002933915.1^30^ | 1, 2 |
| 22 | *Eutrema salsugineum* | Eutsalg1_0 | SC^12^ | GCA_000478725.1^31^ | 1, 2, 3 |
| 23 | *Eutrema yunnanense* | ASM293393v1 | SC^12^ | GCA_002933935.1^30^ | 1, 2 |
| 24 | *Iberis amara* | ASM90040637v1 | SI^13^ | GCA_900406375.1^22^ | 1 |
| 25 | *Kernera saxatilis* | ASM90040639v1 | SI^14^ | GCA_900406395.1^22^ | 1, 2 |
| 26 | *Leavenworthia alabamica* | VEGI_LA_v_1.0 | SC^1^ | GCA_000411055.1^21^ | 1 |
| 27 | *Lepidium africanum* | ASM90040640v1 | SC^15^ | GCA_900406405.1^22^ | 1, 2 |
| 28 | *Lepidium aucheri* | ASM90040643v1 | SI^15^ | GCA_900406435.1^22^ | 1, 2 |
| 29 | *Microthlaspi erraticum* | Merr_genes | SC^16^ | GCA_902728155.2^32^ | 1 |
| 30 | *Physaria fendleri* | ASM90040652v1 | SI^17,19^ | GCA_900406525.1^33^ | 1 |
| 31 | *Pseudoturritis turrita* | ASM90040655v1 | ? | GCA_900406555.1^22^ | 1, 2 |
| 32 | *Raparia bulbosa* | ASM90040653v1 | ? | GCA_900406535.1^22^ | 1, 2 |
| 33 | *Schrenkiella parvula* | Eutrema_parvulum_v01 | SC^1^ | GCA_000218505.1^34^ | 1, 2, 3 |
| 34 | *Sisymbrium altissimum* | ASM90040649v1 | SI^18^ | GCA_900406495.1^22^ | 1, 2 |
| 35 | *Sisymbrium irio* | VEGI_SI_v_1.0 | SC^1^ | GCA_000411075.1^21^ | 1, 2 |
| 36 | *Turritis glabra* | ASM90040656v1 | SC^20^ | GCA_900406565.1^22^ | 1, 2 |

chi_v1 reference link: http://chi.mpipz.mpg.de/assembly.html

References:

1 Busch, 2005

2 Boaz et al., 1990

3 Schmickl et al., 2010

4 Laenen et al., 2018

5 Kudoh, 2016

6 Byrne et al., 2017; Christensen et al., 2016

7 Olowokudejo & Heywood, 1984

8 Rushworth et al., 2011

9 Jiao et al., 2017

10 Zhang et al., 2019

11 Danin & Feinbrun-Dothan, 1991

12 Guo et al., 2018

13 Bateman, 1955

14 Kropf et al., 2006

15 Bowman et al., 1999

16 Mishra et al., 2020

17 Mitchell, 1997

18 Hickey & Clive, 1988

19 Azeez & Bates, 2024

20 *Biodiversity India - India Biodiversity Portal*, 2024

21 Haudry et al., 2013

22 Kiefer et al., 2019

23 Barragan et al., 2024

24 Sailer et al., 2018

25 Chromosome 1, others also available (Theologis et al., 2000)

26 Byrne et al., 2017

27 Kliver et al., 2018

28 Slotte et al., 2013

29 Zhang et al., 2019

30 Guo et al., 2018

31 Yang et al., 2013

32 Mishra et al., 2020

33 Johnston et al., 2024

34 Dassanayake et al., 2011

35 Hu et al., 2011

Tab. S3: Clade assigned for plotting the phylogenetic relationship in Fig. 3, assembly size, estimated genome size based on k-mer frequencies, and the gene number for the used accession.

| Species | Clade | Assembly size  [Mb] | Estimated genome size  [Mb] | Gene number |
| --- | --- | --- | --- | --- |
| *Aethionema arabicum* | root | 203 | 270^1^ | 37839 |
| *Arabidopsis arenosa* | 2 | 150.02 | 179* |  |
| *Arabidopsis halleri* | 2 | 164.6 | 227* |  |
| *Arabis alpina* | 7 | 311.6 | 347^1^ | 23286 |
| *Barbarea vulgaris* | 4 | 246.2 | 270^2^ |  |
| *Boechera retrofracta* | 1 | 216.2 | 227^3^ |  |
| *Boechera stricta* | 1 | 190.5 | 215^1^ |  |
| *Capsella rubella* | 1 | 133.1 | 139* | 28713 |
| *Cardamine hirsuta* MPI | 4 | 198 | 218^1^ | 37997 |
| *Conringia planisiliqua* | 5 | 184.2 | 186^1^ |  |
| *Crucihimalaya himalaica* | 1 | 234.7 | 265^4^ |  |
| *Euclidium syriacum* | 9 | 229.2 | 228^1^ |  |
| *Eutrema heterophyllum* | 5 | 349 | 405^5^ |  |
| *Eutrema salsugineum* | 5 | 243.1 | 258^5^ | 29485 |
| *Eutrema yunnanense* | 5 | 415.4 | 423^5^ |  |
| *Kernera saxatilis* | 6 | 144 | 168^1^ |  |
| *Lepidium africanum* | 3 | 225.3 | 273^1^ |  |
| *Lepidium aucheri* | 3 | 332.1 | 426^1^ |  |
| *Pseudoturritis turrita* | 7 | 321.6 | 321^1^ |  |
| *Raparia bulbosa* | 6 | 152.8 | 203^1^ |  |
| *Schrenkiella parvula* | 5 | 137.1 | 139^1^ | 27132 |
| *Sisymbrium altissimum* | 5 | 178.6 | 211^1^ |  |
| *Sisymbrium irio* | 5 | 245.6 | 258^1^ |  |
| *Turritis glabra* | 1 | 171.1 | 175^1^ |  |
| *Boechera puberula* | 1 | 182.6 | 199^1^ |  |
| *Arabis ciliata* | 7 | 309 | 358* | 33544 |
| *Arabis caerulea* | 7 | 387 | 466* | 36540 |
| *Cardamine resedifolia* | 4 | 245 | 265* | 29697 |
| *Cardamine hirsuta* | 4 | 208 | 229* | 30168 |
| *Noccaea rotundifolia* | 6 | 390 | 383* | 40523 |
| *Noccaea brachypetala* | 6 | 236 | 267* | 36803 |
| *Arabidopsis arenicola* | 2 | 186 | 232* | 31962 |

References:

1) Kiefer et al., 2019

2) Byrne et al., 2017

3) Kliver et al., 2018

4) Zhang et al., 2019

5) Guo et al., 2018

6) Johnston et al., 2024

* own calculations

Tab. S4: Estimation of genome properties, including genome size, average depth of coverage, and the level of heterozygosity, based on the distribution of k-mer frequencies in PacBio HiFi reads.

| Species | Raw peak | Effective k-mer, species | Effective k-mer, individuals | Coverage depth | Genome size [Mb] | K-mer species heterozygous ratio | K-mer individual heterozygous ratio |
| --- | --- | --- | --- | --- | --- | --- | --- |
|  |  |  |  |  |  |  |  |
| *Arabis caerulea* | 15 | 194625966 | 7327636675 | 15.7235 | 466 | 0.0226012 | 0.0062346 |
| *Arabis ciliata* | 20 | 166993295 | 7352622516 | 20.5497 | 358 | 0.0192793 | 0.0050399 |
| *Cardamine resedifolia* | 21 | 133980629 | 5666973559 | 21.4118 | 265 | 0.0177122 | 0.0054334 |
| *Cardamine hirsuta* | 18 | 140385364 | 4264914937 | 18.6102 | 229 | 0.0162809 | 0.0059822 |
| *Noccaea rotundifolia* | 30 | 147923203 | 1060100000 | 27.6637 | 383 | 0.147725 | 0.0299935 |
| *Noccaea brachypetala* | 29 | 123853573 | 7949330063 | 29.8243 | 267 | 0.0551898 | 0.0172465 |
| *Arabidopsis arenicola* | 20 | 123086172 | 4812697226 | 20.7746 | 232 | 0.0176045 | 0.00611663 |

Tab. S5: Distribution of contig sizes and GC content of the new reference genomes. The N50 shows that 50% of each of the assembled genomes was covered by contigs longer than each N50 value (i.e. 8 to 17 Mb). The L50 shows how many of the largest contigs need to be summed up to add up to represent half of the whole genome size. The lower the L50, the higher the quality of the assembly. Bases masked shows the part of the genome consisting of repeated elements, which are therefore masked.

| Species | *A. caerulea* | *A. ciliata* | *C. resedifolia* | *C. hirsuta* | *N. rotundifola* | *N. brachypetala* | *A. arenicola* |
| --- | --- | --- | --- | --- | --- | --- | --- |
| N50 | 11251880 | 17100341 | 11775137 | 11756586 | 8097724 | 12685083 | 11330647 |
| # contigs | 375 | 435 | 335 | 277 | 762 | 1103 | 336 |
| Largest contig | 29815484 | 36767830 | 31721322 | 26572875 | 30410404 | 19913468 | 25363092 |
| Total length | 397820405 | 321646273 | 245691755 | 208767977 | 408043968 | 268721868 | 217874282 |
| GC (%) | 39.77 | 37.77 | 37.83 | 35.55 | 40.93 | 39.65 | 36.86 |
| L50 | 12 | 7 | 7 | 7 | 12 | 8 | 7 |
| Bases masked | 252561583 (63.49 %) | 185846483 (57.78 %) | 123914840 (50.44 %) | 85296351 (40.86 %) | 254019612 (62.25 %) | 133558730 (49.70 %) | 88326682  (40.54 %) |

Tab. S6: Summary statistics of orthogroup inference by OrthoFinder.

| Number of species | 8 |
| --- | --- |
| Number of proteins | 287591 |
| Number of orthogroups | 27100 (19695 have at least one *A. thaliana* protein) |
| Number of species-specific orthogroups | 4142 (1075 are *A. thaliana*-specific) |
| Number of proteins in orthogroups | 259305 |
| Number of single-copy orthogroups | 5418 |

Tab. S7: Median and interquartiles of (absolute term of) latitude, elevation, mean annual temperature (MAT) and precipitation (MAP) across the distribution for the species included in the study (values prior to phylogenetic adjustment). Latitude and elevation for the specimen used to produce the assembly are given in the last two columns, if information was available.

|  | \|Latitude\| [°] | | | Elevation [m a.s.l.] | | | MAT [°C] | | | MAP [mm] | | |  |  |
| --- | --- | --- | --- | --- | --- | --- | --- | --- | --- | --- | --- | --- | --- | --- |
| Species | Median | Q1 | Q3 | Median | Q1 | Q3 | Median | Q1 | Q3 | Median | Q1 | Q3 | Ref. latitude [° N] | Ref. elevation [m a.s.l.] |
| *A. hierochuntica* | 29.64 | 24.62 | 30.88 | 313 | 56 | 745.75 | 23.08 | 17.76 | 28.17 | 80 | 44.5 | 129 |  |  |
| *A. arenicola* | 60.61 | 56.52 | 64.23 | 98 | 11 | 228 | -6.42 | -19.23 | 4.71 | 411 | 280 | 524 | 58.78 | 0 |
| *A. arenosa* | 51.17 | 49.17 | 52.82 | 184 | 63 | 433 | 8.00 | 2.80 | 13.76 | 719 | 602 | 857 | 49.19 | 360 |
| *A. halleri* | 50.67 | 48.42 | 51.17 | 451 | 296.75 | 684 | 7.25 | 1.99 | 13.07 | 814 | 700 | 1048.5 | 46.28 | 780 |
| *A. lyrata* | 42.93 | 40.98 | 45.43 | 250 | 174.5 | 336.5 | 8.20 | -1.02 | 16.30 | 925 | 782.5 | 1132 |  |  |
| *A. thaliana* | 50.17 | 46.80 | 55.46 | 139 | 55 | 339 | 8.50 | 3.77 | 14.18 | 768 | 650 | 928 |  |  |
| *A. alpina* | 56.34 | 45.04 | 65.79 | 637 | 337 | 1019 | 3.26 | -1.72 | 9.69 | 887 | 633 | 1161 | 43.16 | 317 |
| *A. caerulea* | 46.57 | 46.01 | 47.01 | 1984 | 1551 | 2274 | 1.94 | -2.88 | 7.96 | 1277 | 1089 | 1438 | 46.01 | 2670 |
| *A. ciliata* | 46.43 | 44.00 | 47.27 | 1232.5 | 850 | 1776 | 5.57 | 0.96 | 11.61 | 1233 | 1039 | 1339 | 46.01 | 2530 |
| *B. auriculata* | 39.11 | 37.86 | 40.94 | 680 | 383.75 | 845.25 | 13.28 | 9.31 | 20.16 | 451.5 | 405.75 | 550 |  |  |
| *B. longifolia* | 44.87 | 43.60 | 46.95 | 1268 | 800 | 1741 | 6.07 | 1.47 | 12.16 | 1084 | 882.5 | 1320 |  |  |
| *B. divaricarpa* | 43.80 | 41.20 | 45.76 | 1963 | 1526.5 | 2298 | 4.39 | -1.43 | 11.48 | 484 | 358.5 | 648 |  |  |
| *B. puberula* | 41.65 | 39.67 | 42.87 | 1736 | 1471.5 | 1954.25 | 6.95 | 1.40 | 14.12 | 323 | 276.25 | 389.75 |  |  |
| *B. retrofracta* | 44.34 | 40.65 | 49.07 | 1536 | 954 | 2022.5 | 4.82 | -1.67 | 12.17 | 456 | 346 | 636.5 |  |  |
| *B. stricta* | 43.81 | 39.48 | 48.72 | 2068 | 1154 | 2693.5 | 2.94 | -3.88 | 10.43 | 574.5 | 446 | 749 |  |  |
| *B. vulgaris* | 49.97 | 49.32 | 51.15 | 363 | 236 | 460.5 | 8.12 | 3.19 | 13.84 | 776 | 721.5 | 871.5 | 51.41 | 33 |
| *C. himalaica* | 32.63 | 29.17 | 34.50 | 3726 | 2851 | 4190 | 4.66 | -2.02 | 10.73 | 661 | 437 | 843 | 30.31 | 4010 |
| *C. hirsuta* MPI | 48.36 | 45.42 | 52.30 | 176 | 70.25 | 410 | 9.55 | 5.37 | 14.94 | 792 | 687 | 960.75 |  |  |
| *C. hirsuta* | 48.36 | 45.42 | 52.30 | 176 | 70.25 | 410 | 9.55 | 5.37 | 14.94 | 792 | 687 | 960.75 | 47.18 | 415 |
| *C. planisiliqua* | 36.25 | 34.30 | 39.71 | 3202 | 1839 | 4256 | 3.92 | -4.71 | 11.62 | 276 | 177 | 395 |  |  |
| *C. resedifolia* | 46.22 | 43.10 | 46.92 | 1519 | 1159.75 | 1946.25 | 4.66 | 0.04 | 10.73 | 1168 | 955 | 1359.25 | 46.83 | 2645 |
| *C. rubella* | 45.68 | 43.62 | 47.29 | 268 | 126 | 549.75 | 10.80 | 6.61 | 16.40 | 812 | 714 | 998 | 41.73 | 830 |
| *E. syriacum* | 43.55 | 35.75 | 48.70 | 378 | 120 | 1407 | 9.96 | 0.84 | 17.50 | 435 | 304 | 544 |  |  |
| *E. heterophyllum* | 33.93 | 31.13 | 36.98 | 3746 | 2497 | 4492 | 2.64 | -4.96 | 8.98 | 548 | 400 | 646 | 31.40 | 4700 |
| *E. salsugineum* | 53.61 | 51.29 | 56.28 | 406 | 146.25 | 801.5 | 0.48 | -12.80 | 10.61 | 356.5 | 303.5 | 441.75 | 37.43 | 10 |
| *E. yunnanense* | 30.13 | 28.41 | 31.77 | 1474 | 862 | 2381.5 | 12.33 | 6.13 | 17.45 | 907 | 803.5 | 1232 |  |  |
| *I. amara* | 47.55 | 43.88 | 49.72 | 259.5 | 114 | 585.75 | 9.80 | 5.61 | 15.44 | 769 | 662 | 916 |  |  |
| *K. saxatilis* | 46.36 | 43.99 | 47.26 | 1135 | 799 | 1625 | 6.05 | 1.34 | 12.08 | 1174 | 982.5 | 1320 |  |  |
| *L. alabamica* | 34.49 | 34.45 | 34.58 | 201 | 194 | 236 | 15.48 | 9.35 | 22.45 | 1452.5 | 1444 | 1470.75 |  |  |
| *L. africanum* | 31.49 | 26.56 | 33.45 | 201 | 132 | 313.25 | 22.48 | 16.80 | 26.83 | 22 | 1 | 90 |  |  |
| *L. aucheri* | 30.68 | 27.88 | 32.97 | 328 | 269.5 | 695.25 | 21.74 | 14.38 | 27.96 | 103 | 66.75 | 170.5 |  |  |
| *M. erraticum* | 48.13 | 45.81 | 49.37 | 529 | 324 | 681 | 8.37 | 3.10 | 14.33 | 807 | 686 | 1064 |  |  |
| *N. brachypetala* | 44.78 | 43.10 | 45.83 | 1382.5 | 970 | 1877 | 5.51 | 1.07 | 11.65 | 1104.5 | 951 | 1278.5 | 46.92 | 1060 |
| *N. rotundifolia* | 46.56 | 45.31 | 47.25 | 1423.5 | 1072.75 | 1739.5 | 4.83 | 0.02 | 10.84 | 1202.5 | 1009 | 1373 | 47.24 | 2005 |
| *P. fendleri* | 31.89 | 29.50 | 34.10 | 1420 | 1124.25 | 1713 | 15.81 | 9.91 | 21.77 | 358 | 312 | 420 |  |  |
| *P. turrita* | 44.51 | 43.54 | 46.25 | 587 | 320 | 953 | 9.45 | 4.73 | 15.47 | 895 | 744.75 | 1135 |  |  |
| *R. bulbosa* | 38.16 | 37.93 | 38.50 | 408.5 | 358.75 | 523.25 | 14.13 | 8.57 | 20.65 | 611.5 | 512 | 657.75 |  |  |
| *S. altissimum* | 50.00 | 45.63 | 53.79 | 136 | 68.5 | 231 | 7.90 | -1.11 | 15.42 | 505 | 401.25 | 596.75 |  |  |
| *S. irio* | 40.78 | 37.47 | 42.84 | 451.5 | 178.75 | 793.25 | 13.57 | 8.92 | 19.74 | 541.5 | 407 | 732 |  |  |
| *S. parvula* | 49.11 | 46.35 | 49.12 | 19 | 7.5 | 252.5 | 9.83 | -1.08 | 18.82 | 278 | 251.5 | 285.5 |  |  |
| *T. glabra* | 50.93 | 46.47 | 56.12 | 209 | 86 | 486 | 6.91 | 0.78 | 13.40 | 711 | 603 | 908 |  |  |

Tab. S8: Model selection on the dependent variables of genome size, fraction all TE, fraction LTR-TE, and fraction DNA-TE, without or with mating system as cofactor (+ ms). Independent variables (mean-centered) were mean annual temperature (MAT), the square term of MAT, elevation corrected for MAT (residuals of linear regression of elevation on MAT, named res_elev_mat) and its square term (res_elev_mat^2^), mean annual precipitation corrected for MAT (residuals of linear regression of mean annual precipitation on MAT, named res_MAP_MAT) and its square term (res_MAP_MAT^2^), absolute latitude and latitude^2^, N=32. Best models were chosen by AICc (in bold).

| Dependent variable |  | Genome size |  | Fraction tall TE |  | Fraction LTR-TE |  | FractionDNA-TE |  |
| --- | --- | --- | --- | --- | --- | --- | --- | --- | --- |
|  |  |  | + ms |  | + ms |  | + ms |  | +ms |
| Model 1 | R^2^ | -0.03 | -0.06 | 0.02 | -0.01 | 0.06 | 0.03 | 0.00 | -0.01 |
| y ~ MAT | λ | 1 | 1 | 1 | 1 | 1 | 1 | 1 | 1 |
|  | AIC | 352.10 | 353.74 | -31.98 | -30.15 | -50.97 | -49.06 | -137.90 | -136.58 |
|  | AICc | 352.54 | 354.66 | -31.54 | -29.23 | -50.52 | -48.13 | -137.46 | -135.65 |
| Model 2 | R^2^ | 0.10 | 0.08 | 0.22 | 0.19 | 0.16 | 0.13 | 0.02 | 0.00 |
| y ~ MAT + MAT^2^ | λ | 1 | 1 | 1 | 1 | 1 | 1 | 1 | 1 |
|  | AIC | 348.96 | 350.36 | -37.92 | -36.02 | -53.45 | -51.50 | -137.57 | -136.17 |
|  | AICc | 349.88 | 351.96 | -37.00 | -34.42 | -52.53 | -49.90 | -136.65 | -134.57 |
| Model 3 | R^2^ | 0.26 | 0.27 | 0.36 | 0.34 | 0.25 | 0.22 | 0.62 | 0.60 |
| y ~ MAT +MAT^2^ +res_elev_MAT | λ | 1 | 1 | 1 | 1 | 1 | 1 | 1 | 1 |
|  | AIC | 343.89 | 344.23 | -43.15 | -41.15 | -55.68 | -53.69 | -165.03 | -163.11 |
|  | AICc | 345.49 | 346.73 | -41.55 | -38.65 | -54.08 | -51.19 | **-163.43** | -160.61 |
| Model 4 | R^2^ | 0.30 | 0.31 | 0.38 | 0.35 | 0.30 | 0.27 | 0.60 | 0.59 |
| y ~ MAT + MAT^2^ + res_elev_MAT + res_elev_MAT^2^ | λ | 1 | 1 | 1 | 1 | 1 | 1 | 1 | 1 |
|  | AIC | 342.95 | 343.46 | -43.08 | -41.08 | -57.25 | -55.26 | -163.11 | -161.18 |
|  | AICc | 345.45 | 347.11 | -40.58 | -37.43 | -54.75 | -51.61 | -160.61 | -157.53 |
| Model 5 | R^2^ | 0.44 | 0.42 | 0.45 | 0.32 | 0.37 | 0.35 | 0.61 | 0.59 |
| y ~ MAT + MAT^2^ + res_elev_MAT + res_MAP_MAT | λ | 1 | 1 | 1 | 1 | 1 | 1 | 1 | 1 |
|  | AIC | 336.39 | 337.94 | -46.55 | -44.90 | -60.12 | -58.50 | -163.76 | -162.04 |
|  | AICc | **338.89** | 341.59 | **-44.05** | -41.25 | **-57.62** | -54.85 | -161.26 | -158.39 |
| Model 6 | R^2^ | 0.43 | 0.41 | 0.42 | 0.40 | 0.35 | 0.35 | 0.60 | 0.60 |
| y ~ MAT + MAT^2^ + res_elev_MAT + res_MAP_MAT + res_MAP_MAT^2^ | λ | 1 | 1 | 1 | 1 | 1 | 1 | 1 | 1 |
|  | AIC | 337.54 | 339.27 | -44.56 | -42.90 | -58.46 | -56.74 | -162.12 | -160.54 |
|  | AICc | 341.19 | 344.36 | -40.91 | -41.25 | -54.81 | -51.64 | -158.47 | -155.45 |
| Model 7 | R^2^ | 0.08 | 0.05 | -0.01 | -0.04 | -0.02 | -0.04 | 0.46 | 0.46 |
| y ~ \|latitude\| + res_elev_MAT + res_MAP_MAT | λ | 0.87 | 0.911 | 0.92 | 0.90 | 1 | 1 | 0.78 | 0.67 |
|  | AIC | 350.68 | 352.39 | -29.26 | -27.60 | -46.61 | -45.20 | -154.04 | -152.68 |
|  | AICc | 352.28 | 354.89 | -27.66 | -25.10 | -45.01 | -42.70 | -152.44 | -150.18 |
| Model 8 | R^2^ | 0.27 | 0.24 | 0.24 | 0.20 | 0.15 | 0.12 | 0.54 | 0.54 |
| y ~ \|latitude\| + latitude^2^ + res_elev_MAT + res_MAP_MAT | λ | 0.61 | 0.774 | 1 | 1 | 1 | 1 | 0.742 | 0.60 |
|  | AIC | 345.48 | 346.76 | -36.95 | -34.96 | -51.20 | -49.37 | -157.85 | -156.23 |
|  | AICc | 347.98 | 350.42 | -34.45 | -31.31 | -48.70 | -45.71 | -155.35 | -152.58 |

Tab. S9: GO-terms matching expanded gene families in high-elevation species.

| Pval | Description | Legend | Members | Representative |
| --- | --- | --- | --- | --- |
| 7.35E-51 | pectin catabolic process | 1. pectin catabolic process | ['GO:0045488', 'GO:0045490', 'GO:0010393', 'GO:0000272', 'GO:0005976', 'GO:0016052', 'GO:0005975', 'GO:0006007', 'GO:0009057', 'GO:0030163', 'GO:1901575', 'GO:0009056'] | GO:0045490 |
| 1.17E-30 | recognition of pollen | 2. recognition of pollen | ['GO:0008037', 'GO:0048544'] | GO:0048544 |
| 1.01E-29 | protein refolding | 3. protein refolding | ['GO:0006457', 'GO:0042026', 'GO:0061077', 'GO:0051085', 'GO:0051084', 'GO:0006458'] | GO:0042026 |
| 5.60E-24 | innate immune response | 4. innate immune response | ['GO:0006955', 'GO:0045087', 'GO:0002376', 'GO:0140546', 'GO:0098542', 'GO:0006952', 'GO:0044419', 'GO:0009605', 'GO:0043207', 'GO:0006950', 'GO:0050896', 'GO:0051716', 'GO:0009615', 'GO:0051707', 'GO:0009607'] | GO:0045087 |
| 3.64E-19 | protein maturation | 5. protein maturation | ['GO:0051604', 'GO:0006412', 'GO:0043043', 'GO:0006518', 'GO:0009059', 'GO:0043604'] | GO:0051604 |
| 8.95E-18 | protein phosphorylation | 6. protein phosphorylation | ['GO:0006468', 'GO:0018105', 'GO:0036211', 'GO:0018209', 'GO:0043687', 'GO:0043412', 'GO:0006304'] | GO:0006468 |
| 1.31E-13 | cell surface receptor signaling pathway | 7. cell surface receptor signaling pat... | ['GO:0007166', 'GO:0007165', 'GO:0050794', 'GO:0050789', 'GO:0065007'] | GO:0007166 |
| 3.34E-12 | response to temperature stimulus | 8. response to temperature stimulus | ['GO:0009266', 'GO:0009408', 'GO:0009628', 'GO:0010048', 'GO:0034605', 'GO:0071456', 'GO:0036294', 'GO:0071453', 'GO:0001666', 'GO:0036293', 'GO:0070482'] | GO:0009266 |
| 4.11E-11 | negative regulation of flower development | 9. negative regulation of flower devel... | ['GO:0009910', 'GO:0048581', 'GO:0051093', 'GO:2000242', 'GO:2000241', 'GO:0009909', 'GO:0048831', 'GO:0048580', 'GO:0051241', 'GO:0048519', 'GO:0010629', 'GO:2000026', 'GO:0050793', 'GO:0051239'] | GO:0009910 |
| 5.12E-10 | gene expression | 10. gene expression | ['GO:0010467'] | GO:0010467 |
| 6.82E-08 | proton motive force-driven mitochondrial ATP synthesis | 11. proton motive force-driven mitochon... | ['GO:0042776', 'GO:0015986', 'GO:0006754', 'GO:0009206', 'GO:0009145', 'GO:0009201', 'GO:0009142', 'GO:0046034', 'GO:0009205', 'GO:0009144', 'GO:0009199', 'GO:0009141', 'GO:0009150', 'GO:0009259', 'GO:0019693'] | GO:0042776 |
| 2.73E-07 | miRNA transport | 12. miRNA transport | ['GO:1990428', 'GO:0050658', 'GO:0050657', 'GO:0015931', 'GO:0051236'] | GO:1990428 |
| 5.36E-07 | negative regulation of gene express... | 13. negative regulation of gene expression | ['GO:0044027', 'GO:0045814', 'GO:0040029', 'GO:0006338', 'GO:0006325', 'GO:0071824'] | GO:0044027 |
| 4.53E-06 | regulation of transcription by RNA polymerase II | 14. regulation of transcription by RNA ... | ['GO:0006357', 'GO:0045944', 'GO:0006355', 'GO:2001141', 'GO:0051252', 'GO:0019219', 'GO:0051171', 'GO:0080090', 'GO:0010468', 'GO:0010556', 'GO:0031326', 'GO:0009889', 'GO:0031323', 'GO:0060255'] | GO:0006357 |
| 1.59E-05 | xenobiotic detoxification by transmembrane export across the plasma membrane | 15. xenobiotic detoxification by transm... | ['GO:0046618', 'GO:1990961', 'GO:0042908', 'GO:0140115', 'GO:0140352'] | GO:1990961 |
| 3.55E-05 | oxidative phosphorylation | 16. oxidative phosphorylation | ['GO:0006119', 'GO:0009060', 'GO:0045333', 'GO:0015980', 'GO:0006091'] | GO:0006119 |
| 4.44E-05 | translational initiation | 17. translational initiation | ['GO:0006413'] | GO:0006413 |
| 5.20E-05 | phosphorylation | 18. phosphorylation | ['GO:0016310', 'GO:0006796', 'GO:0006793', 'GO:0019637', 'GO:0006163', 'GO:0072521', 'GO:1901564', 'GO:0006807', 'GO:0009117', 'GO:0006753', 'GO:0055086'] | GO:0016310 |
| 0.00012 | endoplasmic reticulum unfolded protein response | 19. endoplasmic reticulum unfolded prot... | ['GO:0030968', 'GO:0034976', 'GO:0036503'] | GO:0030968 |
| 0.00017 | purine ribonucleotide biosynthetic process | 20. purine ribonucleotide biosynthetic ... | ['GO:0009152', 'GO:0009260', 'GO:0046390', 'GO:1901137', 'GO:0006164', 'GO:0072522', 'GO:0009165', 'GO:1901293', 'GO:0034654', 'GO:0090407', 'GO:0044249', 'GO:0044237', 'GO:1901576', 'GO:0009058'] | GO:0009152 |
| 0.00018 | cellular response to unfolded protein | 21. cellular response to unfolded prote... | ['GO:0034620', 'GO:0035967', 'GO:0006986', 'GO:0035966', 'GO:0009636'] | GO:0034620 |
| 0.00027 | extracellular transport | 22. extracellular transport | ['GO:0006858'] | GO:0006858 |
| 0.00057 | detoxification | 23. detoxification | ['GO:0098754'] | GO:0098754 |
| 0.00061 | root development | 24. root development | ['GO:0048364', 'GO:0099402'] | GO:0048364 |
| 0.00062 | root system development | 25. root system development | ['GO:0022622', 'GO:0048367'] | GO:0022622 |
| 0.00066 | protein ubiquitination | 26. protein ubiquitination | ['GO:0016567', 'GO:0032446', 'GO:0070647', 'GO:0006506', 'GO:0006497', 'GO:0006505', 'GO:0006661', 'GO:0046474', 'GO:0046488', 'GO:0009247', 'GO:0006664', 'GO:0046467', 'GO:0006643', 'GO:1903509'] | GO:0016567 |
| 0.00089 | protein transport to vacuole involved in ubiquitin-dependent protein catabolic process | 27. protein transport to vacuole involv... | ['GO:0043328', 'GO:0032511', 'GO:0032509', 'GO:0016197', 'GO:0071985', 'GO:0045324', 'GO:0007034', 'GO:0072666', 'GO:0072665', 'GO:0006406', 'GO:0006405', 'GO:0051028', 'GO:0051168', 'GO:0006913', 'GO:0051169'] | GO:0043328 |
| 0.0013 | ubiquitin-dependent protein catabolic process | 28. ubiquitin-dependent protein catabol... | ['GO:0043162', 'GO:0051603', 'GO:0006508', 'GO:0019538', 'GO:0043170', 'GO:0071704', 'GO:0008152'] | GO:0043162 |
| 0.0025 | DNA methylation on cytosine | 29. DNA methylation on cytosine | ['GO:0006306', 'GO:0032776', 'GO:0006305'] | GO:0032776 |
| 0.0025 | alternative mRNA splicing, via spliceosome | 30. alternative mRNA splicing, via spli... | ['GO:0000380', 'GO:0045292'] | GO:0000380 |
| 0.0041 | primary metabolic process | 31. primary metabolic process | ['GO:0044238'] | GO:0044238 |
| 0.0053 | regulation of defense response to virus | 32. regulation of defense response to v... | ['GO:0034052', 'GO:0050688'] | GO:0050688 |
| 0.0061 | signaling | 33. signaling | ['GO:0023052'] | GO:0023052 |
| 0.0064 | RNA localization | 34. RNA localization | ['GO:0006403'] | GO:0006403 |
| 0.011 | cell communication | 35. cell communication | ['GO:0007154'] | GO:0007154 |
| 0.018 | lipoprotein biosynthetic process | 36. lipoprotein biosynthetic process | ['GO:0042157', 'GO:0042158'] | GO:0042158 |

Tab. S10: GO-terms matching contracted gene families in high-elevation species.

| pval | Description | Legend | Members | Representative |
| --- | --- | --- | --- | --- |
| 1.22E-52 | sesquiterpene biosynthetic process | 1. sesquiterpene biosynthetic process | ['GO:0051761', 'GO:0051762', 'GO:0046246', 'GO:0042214', 'GO:0120251', 'GO:0120252'] | GO:0051762 |
| 7.35E-51 | pectin catabolic process | 2. pectin catabolic process | ['GO:0000272', 'GO:0045490', 'GO:0045488', 'GO:0010393', 'GO:0045489', 'GO:0052546'] | GO:0045490 |
| 5.20E-42 | carbohydrate catabolic process | 3. carbohydrate catabolic process | ['GO:0005975', 'GO:0016052', 'GO:0006007', 'GO:1901575', 'GO:0009056', 'GO:1901565'] | GO:0016052 |
| 7.20E-41 | SCF-dependent proteasomal ubiquitin-dependent protein catabolic process | 4. SCF-dependent proteasomal ubiquitin... | ['GO:0031146', 'GO:0043161', 'GO:0010498', 'GO:0030163'] | GO:0031146 |
| 2.02E-37 | proton export across plasma membrane | 5. proton export across plasma membran... | ['GO:0120029', 'GO:0140115', 'GO:0140352'] | GO:0120029 |
| 2.62E-33 | regulation of intracellular pH | 6. regulation of intracellular pH | ['GO:0030641', 'GO:0051453', 'GO:0006885', 'GO:0030003', 'GO:0006873', 'GO:0055080', 'GO:0050801'] | GO:0051453 |
| 4.63E-31 | farnesyl diphosphate biosynthetic process | 7. farnesyl diphosphate biosynthetic p... | ['GO:0016106', 'GO:0045337', 'GO:0006714', 'GO:0033384', 'GO:0033383', 'GO:0033386', 'GO:0033385', 'GO:0045338'] | GO:0045337 |
| 1.37E-28 | triglyceride biosynthetic process | 8. triglyceride biosynthetic process | ['GO:0006641', 'GO:0019432', 'GO:0006639', 'GO:0006638', 'GO:0046463', 'GO:0046460'] | GO:0019432 |
| 1.93E-28 | proton transmembrane transport | 9. proton transmembrane transport | ['GO:0098655', 'GO:1902600', 'GO:0006812', 'GO:0006811', 'GO:0030001', 'GO:0034220', 'GO:0098660', 'GO:0070588', 'GO:0006816', 'GO:0098662'] | GO:1902600 |
| 5.60E-24 | innate immune response | 10. innate immune response | ['GO:0006955', 'GO:0045087', 'GO:0002376', 'GO:0140546', 'GO:0051707', 'GO:0043207', 'GO:0009605', 'GO:0009607', 'GO:0044419'] | GO:0045087 |
| 7.60E-24 | defense response to bacterium | 11. defense response to bacterium | ['GO:0009617', 'GO:0042742', 'GO:0098542', 'GO:0006952'] | GO:0042742 |
| 2.59E-20 | glycerolipid biosynthetic process | 12. glycerolipid biosynthetic process | ['GO:0045017', 'GO:0046486'] | GO:0045017 |
| 5.76E-19 | phospholipid biosynthetic process | 13. phospholipid biosynthetic process | ['GO:0006644', 'GO:0008654', 'GO:0008610', 'GO:0044255', 'GO:0006629'] | GO:0008654 |
| 4.53E-17 | peptidyl-serine phosphorylation | 14. peptidyl-serine phosphorylation | ['GO:0018105', 'GO:0018209', 'GO:0046777', 'GO:0006468'] | GO:0018105 |
| 1.33E-16 | protein polyubiquitination | 15. protein polyubiquitination | ['GO:0000209', 'GO:0016567'] | GO:0000209 |
| 6.35E-16 | protein peptidyl-prolyl isomerization | 16. protein peptidyl-prolyl isomerizati... | ['GO:0000413', 'GO:0018208'] | GO:0000413 |
| 4.32E-14 | phospholipid translocation | 17. phospholipid translocation | ['GO:0015914', 'GO:0045332', 'GO:0006869', 'GO:0015748', 'GO:0034204', 'GO:0097035', 'GO:0061024'] | GO:0045332 |
| 6.61E-14 | glucosinolate biosynthetic process | 18. glucosinolate biosynthetic process | ['GO:0016144', 'GO:0019761', 'GO:0019758', 'GO:0019762', 'GO:0016145', 'GO:0019759', 'GO:0019760', 'GO:0016143', 'GO:0019757'] | GO:0019761 |
| 2.05E-13 | glycosyl compound biosynthetic process | 19. glycosyl compound biosynthetic proc... | ['GO:1901657', 'GO:1901659', 'GO:1901658', 'GO:0009152', 'GO:0009260', 'GO:0046390', 'GO:1901137', 'GO:1901135'] | GO:1901659 |
| 1.08E-12 | intracellular auxin homeostasis | 20. intracellular auxin homeostasis | ['GO:0140964', 'GO:0055082', 'GO:0048878', 'GO:0051480', 'GO:0055081', 'GO:0019725', 'GO:0042592'] | GO:0140964 |
| 1.37E-12 | sulfate transport | 21. sulfate transport | ['GO:0008272', 'GO:0072348', 'GO:1902358'] | GO:0008272 |
| 2.26E-12 | intracellular signal transduction | 22. intracellular signal transduction | ['GO:0007165', 'GO:0035556', 'GO:0019722', 'GO:0050794'] | GO:0035556 |
| 4.80E-12 | ubiquitin-dependent protein catabolic process | 23. ubiquitin-dependent protein catabol... | ['GO:0006511', 'GO:0019941', 'GO:0043632', 'GO:0009057', 'GO:0051603'] | GO:0006511 |
| 1.49E-11 | glycogen biosynthetic process | 24. glycogen biosynthetic process | ['GO:0005978', 'GO:0009250', 'GO:0019252', 'GO:0005982'] | GO:0005978 |
| 2.35E-11 | regulation of transcription by RNA polymerase II | 25. regulation of transcription by RNA ... | ['GO:0006357', 'GO:0045944'] | GO:0006357 |
| 3.76E-11 | diterpenoid biosynthetic process | 26. diterpenoid biosynthetic process | ['GO:0016101', 'GO:0016102', 'GO:0016114', 'GO:0006721', 'GO:0008299', 'GO:0006720', 'GO:0016099', 'GO:0016098'] | GO:0016102 |
| 5.07E-11 | regulation of protein localization | 27. regulation of protein localization ... | ['GO:2000008', 'GO:0032880', 'GO:0060341', 'GO:0032879'] | GO:2000008 |
| 9.03E-11 | regulation of double fertilization forming a zygote and endosperm | 28. regulation of double fertilization ... | ['GO:0080154', 'GO:0080155', 'GO:2000241', 'GO:0080092'] | GO:0080155 |
| 1.57E-10 | proteolysis | 29. proteolysis | ['GO:0006508', 'GO:0019538', 'GO:0051604'] | GO:0006508 |
| 1.62E-10 | glycogen metabolic process | 30. glycogen metabolic process | ['GO:0005977', 'GO:0006112', 'GO:0044042'] | GO:0005977 |
| 2.15E-10 | protein modification by small protein conjugation or removal | 31. protein modification by small prote... | ['GO:0032446', 'GO:0070647', 'GO:0043687', 'GO:0043412'] | GO:0032446 |
| 2.28E-10 | recognition of pollen | 32. recognition of pollen | ['GO:0008037', 'GO:0048544'] | GO:0048544 |
| 5.91E-10 | sulfur compound biosynthetic process | 33. sulfur compound biosynthetic proces... | ['GO:0006790', 'GO:0044272', 'GO:0006749', 'GO:0044273'] | GO:0044272 |
| 7.97E-10 | double fertilization forming a zygote and endosperm | 34. double fertilization forming a zygo... | ['GO:0009566', 'GO:0009567', 'GO:0022414', 'GO:0003006'] | GO:0009567 |
| 1.37E-09 | peptidyl-amino acid modification | 35. peptidyl-amino acid modification | ['GO:0018193', 'GO:0036211'] | GO:0018193 |
| 2.21E-09 | inorganic anion transport | 36. inorganic anion transport | ['GO:0015698', 'GO:0098661'] | GO:0015698 |
| 6.20E-09 | mucilage biosynthetic process | 37. mucilage biosynthetic process | ['GO:0010191', 'GO:0010192'] | GO:0010192 |
| 2.55E-08 | methylation | 38. methylation | ['GO:0032259'] | GO:0032259 |
| 4.68E-08 | seed coat development | 39. seed coat development | ['GO:0010214', 'GO:0048316', 'GO:0009791'] | GO:0010214 |
| 4.75E-08 | secondary metabolic process | 40. secondary metabolic process | ['GO:0009407', 'GO:0019748', 'GO:0009404', 'GO:0009809', 'GO:0009699', 'GO:0009698', 'GO:0009808', 'GO:0044550'] | GO:0019748 |
| 6.82E-08 | proton motive force-driven mitochondrial ATP synthesis | 41. proton motive force-driven mitochon... | ['GO:0042776', 'GO:0015986', 'GO:0006754', 'GO:0009206', 'GO:0009145', 'GO:0009201', 'GO:0009142'] | GO:0042776 |
| 6.63E-07 | signaling | 42. signaling | ['GO:0023052'] | GO:0023052 |
| 6.72E-07 | sexual reproduction | 43. sexual reproduction | ['GO:0000003', 'GO:0019953'] | GO:0019953 |
| 8.75E-07 | lipid localization | 44. lipid localization | ['GO:0010876'] | GO:0010876 |
| 1.38E-06 | endoplasmic reticulum unfolded protein response | 45. endoplasmic reticulum unfolded prot... | ['GO:0030968', 'GO:0034976'] | GO:0030968 |
| 1.84E-06 | cell communication | 46. cell communication | ['GO:0007154', 'GO:0007267'] | GO:0007154 |
| 2.38E-06 | response to unfolded protein | 47. response to unfolded protein | ['GO:0006986', 'GO:0035966', 'GO:0009733', 'GO:0009751'] | GO:0006986 |
| 2.38E-06 | cellular response to unfolded protein | 48. cellular response to unfolded prote... | ['GO:0034620', 'GO:0035967', 'GO:0071333', 'GO:0071331', 'GO:0071326'] | GO:0034620 |
| 2.66E-06 | DNA duplex unwinding | 49. DNA duplex unwinding | ['GO:0032392', 'GO:0032508', 'GO:0071103', 'GO:0051276'] | GO:0032508 |
| 2.97E-06 | protein stabilization | 50. protein stabilization | ['GO:0031647', 'GO:0050821', 'GO:0065008', 'GO:0010817'] | GO:0050821 |
| 3.09E-06 | regulation of DNA-templated transcription | 51. regulation of DNA-templated transcr... | ['GO:0006355', 'GO:2001141', 'GO:0051252', 'GO:0019219', 'GO:0051171', 'GO:0080090'] | GO:0006355 |
| 3.65E-06 | salicylic acid metabolic process | 52. salicylic acid metabolic process | ['GO:0009696', 'GO:0018958', 'GO:0042537', 'GO:1901615'] | GO:0009696 |
| 3.69E-06 | protein localization involved in autophagy | 53. protein localization involved in au... | ['GO:1901703'] | GO:1901703 |
| 4.67E-06 | response to stress | 54. response to stress | ['GO:0006950', 'GO:0050896', 'GO:0051716'] | GO:0006950 |
| 6.85E-06 | siRNA processing | 55. siRNA processing | ['GO:0030422', 'GO:0070918', 'GO:0034470'] | GO:0030422 |
| 6.85E-06 | polysaccharide biosynthetic process | 56. polysaccharide biosynthetic process | ['GO:0000271', 'GO:0005976', 'GO:0016051'] | GO:0000271 |
| 7.42E-06 | arginyl-tRNA aminoacylation | 57. arginyl-tRNA aminoacylation | ['GO:0006420', 'GO:0006418', 'GO:0043039', 'GO:0043038'] | GO:0006420 |
| 1.50E-05 | jasmonic acid metabolic process | 58. jasmonic acid metabolic process | ['GO:0001676', 'GO:0009694', 'GO:0006631', 'GO:0032787'] | GO:0009694 |
| 1.74E-05 | regulation of gene expression | 59. regulation of gene expression | ['GO:0010468', 'GO:0010556', 'GO:0031326', 'GO:0009889', 'GO:0031323', 'GO:0019222', 'GO:0060255'] | GO:0010468 |
| 3.55E-05 | oxidative phosphorylation | 60. oxidative phosphorylation | ['GO:0006119', 'GO:0009060', 'GO:0045333', 'GO:0015980', 'GO:0006091'] | GO:0006119 |
| 5.93E-05 | aromatic compound biosynthetic process | 61. aromatic compound biosynthetic proc... | ['GO:0006725', 'GO:0019438', 'GO:0034654'] | GO:0019438 |
| 7.42E-05 | CTP salvage | 62. CTP salvage | ['GO:0044211', 'GO:0044206', 'GO:0010138', 'GO:0032262', 'GO:0008655', 'GO:0043173', 'GO:0043094'] | GO:0044211 |
| 8.65E-05 | cellular response to heat | 63. cellular response to heat | ['GO:0009408', 'GO:0034605', 'GO:0009266', 'GO:0070482'] | GO:0034605 |
| 9.86E-05 | ATP metabolic process | 64. ATP metabolic process | ['GO:0009205', 'GO:0046034', 'GO:0009144', 'GO:0009141', 'GO:0009199'] | GO:0046034 |
| 0.00021ß | primary miRNA processing | 65. primary miRNA processing | ['GO:0031053', 'GO:0035196'] | GO:0031053 |
| 0.00024 | oxoacid metabolic process | 66. oxoacid metabolic process | ['GO:0006082', 'GO:0043436', 'GO:0044281'] | GO:0043436 |
| 0.00026 | DNA replication | 67. DNA replication | ['GO:0006259', 'GO:0006260', 'GO:0034660'] | GO:0006260 |
| 0.00028 | purine nucleotide biosynthetic proces | 68. purine nucleotide biosynthetic proc... | ['GO:0006164', 'GO:0072522'] | GO:0006164 |
| 0.00030 | regulatory ncRNA-mediated gene silencing | 69. regulatory ncRNA-mediated gene sile... | ['GO:0031047', 'GO:0010629', 'GO:0010558', 'GO:0031327', 'GO:0009890'] | GO:0031047 |
| 0.00032 | gene expression | 70. gene expression | ['GO:0010467'] | GO:0010467 |
| 0.00033 | selenate transport | 71. selenate transport | ['GO:0080160'] | GO:0080160 |
| 0.00035 | wax biosynthetic process | 72. wax biosynthetic process | ['GO:0010025', 'GO:0010166', 'GO:1901570', 'GO:1901568'] | GO:0010025 |
| 0.00041 | UMP biosynthetic process | 73. UMP biosynthetic process | ['GO:0006222', 'GO:0046049', 'GO:0009174', 'GO:0009130', 'GO:0009129', 'GO:0009173', 'GO:0009220', 'GO:0009218'] | GO:0006222 |
| 0.00044 | protein folding | 74. protein folding | ['GO:0006457'] | GO:0006457 |
| 0.00046 | auxin polar transport | 75. auxin polar transport | ['GO:0009926', 'GO:0060918', 'GO:0009914'] | GO:0009926 |
| 0.00053 | cellular response to hypoxia | 76. cellular response to hypoxia | ['GO:0036294', 'GO:0071456', 'GO:0071453', 'GO:0001666', 'GO:0036293'] | GO:0071456 |
| 0.00063 | nucleotide biosynthetic process | 77. nucleotide biosynthetic process | ['GO:0009165', 'GO:1901293', 'GO:0090407'] | GO:0009165 |
| 0.00067 | guard cell morphogenesis | 78. guard cell morphogenesis | ['GO:0010442'] | GO:0010442 |
| 0.00098 | macromolecule localization | 79. macromolecule localization | ['GO:0033036', 'GO:0051179'] | GO:0033036 |
| 0.0011 | purine ribonucleotide metabolic process | 80. purine ribonucleotide metabolic pro... | ['GO:0009150', 'GO:0009259', 'GO:0019693'] | GO:0009150 |
| 0.0011 | guard cell development | 81. guard cell development | ['GO:0010441'] | GO:0010441 |
| 0.0011 | CTP biosynthetic process | 82. CTP biosynthetic process | ['GO:0006241', 'GO:0046036', 'GO:0009209', 'GO:0009148', 'GO:0009147', 'GO:0009208'] | GO:0006241 |
| 0.0012 | organic substance transport | 83. organic substance transport | ['GO:0071702', 'GO:0055085', 'GO:0006810', 'GO:0051234'] | GO:0071702 |
| 0.0012 | macromolecule biosynthetic process | 84. macromolecule biosynthetic process | ['GO:0009059', 'GO:0044249', 'GO:0043170'] | GO:0009059 |
| 0.0016 | organic substance metabolic process | 85. organic substance metabolic process | ['GO:0008152', 'GO:0071704', 'GO:0044237', 'GO:0009987', 'GO:0044238', 'GO:1901564', 'GO:0006807'] | GO:0071704 |
| 0.0020 | fruit development | 86. fruit development | ['GO:0010154', 'GO:0048608'] | GO:0010154 |
| 0.0026 | spliceosomal complex assembly | 87. spliceosomal complex assembly | ['GO:0000245', 'GO:0051131'] | GO:0000245 |
| 0.0028 | purine nucleotide metabolic process | 88. purine nucleotide metabolic process | ['GO:0006163', 'GO:0072521'] | GO:0006163 |
| 0.0032 | organonitrogen compound biosynthetic process | 89. organonitrogen compound biosyntheti... | ['GO:1901566', 'GO:1901576', 'GO:0009058'] | GO:1901566 |
| 0.0039 | pyrimidine nucleotide biosynthetic process | 90. pyrimidine nucleotide biosynthetic ... | ['GO:0006220', 'GO:0006221', 'GO:0072528', 'GO:0072527'] | GO:0006221 |
| 0.0044 | programmed cell death | 91. programmed cell death | ['GO:0008219', 'GO:0012501'] | GO:0012501 |
| 0.0045 | nucleotide metabolic process | 92. nucleotide metabolic process | ['GO:0006753', 'GO:0009117', 'GO:0055086'] | GO:0009117 |
| 0.0046 | regulation of biological process | 93. regulation of biological process | ['GO:0050789', 'GO:0065007'] | GO:0050789 |
| 0.0047 | retrograde vesicle-mediated transport, Golgi to ER | 94. retrograde vesicle-mediated transpo... | ['GO:0006888', 'GO:0006890', 'GO:0006891'] | GO:0006890 |
| 0.0050 | mucilage pectin biosynthetic process | 95. mucilage pectin biosynthetic proces... | ['GO:0048358', 'GO:0048363'] | GO:0048358 |
| 0.0056 | oxylipin biosynthetic process | 96. oxylipin biosynthetic process | ['GO:0031407', 'GO:0031408'] | GO:0031408 |
| 0.0061 | ribonucleoside monophosphate biosynthetic process | 97. ribonucleoside monophosphate biosyn... | ['GO:0009124', 'GO:0009156', 'GO:0009123', 'GO:0009161'] | GO:0009156 |
| 0.0065 | phosphorylation | 98. phosphorylation | ['GO:0016310', 'GO:0006796', 'GO:0006793', 'GO:0019637'] | GO:0016310 |
| 0.0067 | regulation of jasmonic acid mediated signaling pathway | 99. regulation of jasmonic acid mediate... | ['GO:2000022'] | GO:2000022 |
| 0.0089 | abscisic acid-activated signaling pathway | 100. abscisic acid-activated signaling p... | ['GO:0009738'] | GO:0009738 |
| 0.013 | plant-type cell wall organization | 101. plant-type cell wall organization | ['GO:0009664'] | GO:0009664 |
| 0.013 | reproductive system development | 102. reproductive system development | ['GO:0061458'] | GO:0061458 |
| 0.014 | positive regulation of plant-type hypersensitive respons | 103. positive regulation of plant-type h... | ['GO:0034052', 'GO:0043069'] | GO:0034052 |
| 0.016 | cellular component organization | 104. cellular component organization | ['GO:0016043', 'GO:0071840'] | GO:0016043 |
| 0.017 | positive regulation of DNA-templated transcription | 105. positive regulation of DNA-template... | ['GO:0045893', 'GO:1902680', 'GO:0051254', 'GO:0045935'] | GO:0045893 |
| 0.021 | response to herbivore | 106. response to herbivore | ['GO:0080027'] | GO:0080027 |
| 0.023 | cellular response to abscisic acid stimulus | 107. cellular response to abscisic acid ... | ['GO:0071215', 'GO:0097306'] | GO:0071215 |
| 0.027 | negative regulation of macromolecule metabolic process | 108. negative regulation of macromolecul... | ['GO:0010605', 'GO:0031324', 'GO:0009892'] | GO:0010605 |
| 0.030 | response to water deprivation | 109. response to water deprivation | ['GO:0009414', 'GO:0009415', 'GO:0001101'] | GO:0009414 |
| 0.033 | positive regulation of macromolecule metabolic process | 110. positive regulation of macromolecul... | ['GO:0010557', 'GO:0031328', 'GO:0009891'] | GO:0010557 |
| 0.033 | guard cell differentiation | 111. guard cell differentiation | ['GO:0010052'] | GO:0010052 |

**Commands used for bioinformatic analyses**

Reference genome assembly

| Step | Command |
| --- | --- |
| Assembly using hifiasm | hifiasm -o bc1008_v152l3 -l3 -t 8 bc1008.fastq.gz  hifiasm -o bc1009_v152l3 -l3 -t 8 bc1009.fastq.gz  hifiasm -o bc1010_v152l3 -l3 -t 8 bc1010.fastq.gz demultiplex.bc1010_BAK8A_OA--bc1010_BAK8A_OA.hifi_reads.fastq.gz  hifiasm -o bc1011_v152l3 -l3 -t 8 bc1011.fastq.gz demultiplex.bc1011_BAK8A_OA--bc1011_BAK8A_OA.hifi_reads.fastq.gz  hifiasm -o bc1012_v152l3 -l3 -t 8 bc1012.fastq.gz  hifiasm -o bc1020_v152l3 -l3 -t 8 bc1020.fastq.gz  hifiasm -o bc1021_v152l3 -l3 -t 8 bc1021.fastq.gz |
| Quast | usr/local/ngseq/packages/QC/QUAST/5.0.2/quast -o quast_primary bc1008_v152l3.bp.p_ctg.fa bc1009_v152l3.bp.p_ctg.fa bc1010_v152l3.bp.p_ctg.fa bc1011_v152l3.bp.p_ctg.fa bc1012_v152l3.bp.p_ctg.fa bc1020  _v152l3.bp.p_ctg.fa bc1021_v152l3.bp.p_ctg.fa |
| Busco | busco -i bc1008_v152l3.bp.p_ctg.fa -l viridiplantae_odb10 -o busco_bc1008_v152l3_pctg -m genome &> scripts/busco_bc1008_v152l3_pctg.log |
| Kmer analysis  GCE prep | #Kmer indivdual number: 7502804231  #Kmer indivdual number: 7446589391  #Kmer indivdual number: 10747828656  #Kmer indivdual number: 8069765743  #Kmer indivdual number: 4925971251  #Kmer indivdual number: 5778025582  #Kmer indivdual number: 4345596066  /usr/local/ngseq/src/GCE/gce-1.0.2/gce -g 7502804231 -f bc1008.AF.kmer.freq.stat.2colum -c 20 -H 1 >bc1008.het.gce.table 2>bc1008.het.gce.log  /usr/local/ngseq/src/GCE/gce-1.0.2/gce -g 7446589391 -f bc1009.AF.kmer.freq.stat.2colum -c 15 -H 1 >bc1009.het.gce.table 2>bc1009.het.gce.log  /usr/local/ngseq/src/GCE/gce-1.0.2/gce -g 10747828656 -f bc1010.AF.kmer.freq.stat.2colum -c 27 -H 1 >bc1010.het.gce.table 2>bc1010.het.gce.log  /usr/local/ngseq/src/GCE/gce-1.0.2/gce -g 8069765743 -f bc1011.AF.kmer.freq.stat.2colum -c 29 -H 1 >bc1011.het.gce.table 2>bc1011.het.gce.log  /usr/local/ngseq/src/GCE/gce-1.0.2/gce -g 4925971251 -f bc1012.AF.kmer.freq.stat.2colum -c 20 -H 1 >bc1012.het.gce.table 2>bc1012.het.gce.log  /usr/local/ngseq/src/GCE/gce-1.0.2/gce -g 5778025582 -f bc1020.AF.kmer.freq.stat.2colum -c 21 -H 1 >bc1020.het.gce.table 2>bc1020.het.gce.log  /usr/local/ngseq/src/GCE/gce-1.0.2/gce -g 4345596066 -f bc1021.AF.kmer.freq.stat.2colum -c 18 -H 1 >bc1021.het.gce.table 2>bc1021.het.gce.log |
| Kmer analysis  GCE het | #Kmer indivdual number: 7502804231  #Kmer indivdual number: 7446589391  #Kmer indivdual number: 10747828656  #Kmer indivdual number: 8069765743  #Kmer indivdual number: 4925971251  #Kmer indivdual number: 5778025582  #Kmer indivdual number: 4345596066  /usr/local/ngseq/src/GCE/gce-1.0.2/gce -g 7502804231 -f bc1008.AF.kmer.freq.stat.2colum >bc1008.gce.table 2>bc1008.gce.log  /usr/local/ngseq/src/GCE/gce-1.0.2/gce -g 7446589391 -f bc1009.AF.kmer.freq.stat.2colum >bc1009.gce.table 2>bc1009.gce.log  /usr/local/ngseq/src/GCE/gce-1.0.2/gce -g 10747828656 -f bc1010.AF.kmer.freq.stat.2colum >bc1010.gce.table 2>bc1010.gce.log  /usr/local/ngseq/src/GCE/gce-1.0.2/gce -g 8069765743 -f bc1011.AF.kmer.freq.stat.2colum >bc1011.gce.table 2>bc1011.gce.log  /usr/local/ngseq/src/GCE/gce-1.0.2/gce -g 4925971251 -f bc1012.AF.kmer.freq.stat.2colum >bc1012.gce.table 2>bc1012.gce.log  /usr/local/ngseq/src/GCE/gce-1.0.2/gce -g 5778025582 -f bc1020.AF.kmer.freq.stat.2colum >bc1020.gce.table 2>bc1020.gce.log  /usr/local/ngseq/src/GCE/gce-1.0.2/gce -g 4345596066 -f bc1021.AF.kmer.freq.stat.2colum >bc1021.gce.table 2>bc1021.gce.log |
| Kmer analysis  GCE | #Kmer indivdual number: 7502804231  #Kmer indivdual number: 7446589391  #Kmer indivdual number: 10747828656  #Kmer indivdual number: 8069765743  #Kmer indivdual number: 4925971251  #Kmer indivdual number: 5778025582  #Kmer indivdual number: 4345596066  /usr/local/ngseq/src/GCE/gce-1.0.2/gce -g 7502804231 -f bc1008.AF.kmer.freq.stat.2colum >bc1008.gce.table 2>bc1008.gce.log  /usr/local/ngseq/src/GCE/gce-1.0.2/gce -g 7446589391 -f bc1009.AF.kmer.freq.stat.2colum >bc1009.gce.table 2>bc1009.gce.log  /usr/local/ngseq/src/GCE/gce-1.0.2/gce -g 10747828656 -f bc1010.AF.kmer.freq.stat.2colum >bc1010.gce.table 2>bc1010.gce.log  /usr/local/ngseq/src/GCE/gce-1.0.2/gce -g 8069765743 -f bc1011.AF.kmer.freq.stat.2colum >bc1011.gce.table 2>bc1011.gce.log  /usr/local/ngseq/src/GCE/gce-1.0.2/gce -g 4925971251 -f bc1012.AF.kmer.freq.stat.2colum >bc1012.gce.table 2>bc1012.gce.log  /usr/local/ngseq/src/GCE/gce-1.0.2/gce -g 5778025582 -f bc1020.AF.kmer.freq.stat.2colum >bc1020.gce.table 2>bc1020.gce.log  /usr/local/ngseq/src/GCE/gce-1.0.2/gce -g 4345596066 -f bc1021.AF.kmer.freq.stat.2colum >bc1021.gce.table 2>bc1021.gce.log |
| Kmer analysis  Kmerfreq | /usr/local/ngseq/src/kmerfreq/kmerfreq -k 17 -t 8 -f 2 -w 1 -c 1 -q 1 -p bc1008 bc1008.lib 2> bc1008.kmerfreq.log  /usr/local/ngseq/src/kmerfreq/kmerfreq -k 17 -t 8 -f 2 -w 1 -c 1 -q 1 -p bc1009 bc1009.lib 2> bc1009.kmerfreq.log  /usr/local/ngseq/src/kmerfreq/kmerfreq -k 17 -t 8 -f 2 -w 1 -c 1 -q 1 -p bc1010 bc1010.lib 2> bc1010.kmerfreq.log  /usr/local/ngseq/src/kmerfreq/kmerfreq -k 17 -t 8 -f 2 -w 1 -c 1 -q 1 -p bc1011 bc1011.lib 2> bc1011.kmerfreq.log  /usr/local/ngseq/src/kmerfreq/kmerfreq -k 17 -t 8 -f 2 -w 1 -c 1 -q 1 -p bc1012 bc1012.lib 2> bc1012.kmerfreq.log  /usr/local/ngseq/src/kmerfreq/kmerfreq -k 17 -t 8 -f 2 -w 1 -c 1 -q 1 -p bc1020 bc1020.lib 2> bc1020.kmerfreq.log  /usr/local/ngseq/src/kmerfreq/kmerfreq -k 17 -t 8 -f 2 -w 1 -c 1 -q 1 -p bc1021 bc1021.lib 2> bc1021.kmerfreq.log |

Annotation with Augustus

| Training (example for one species) | #get list of genes with 100% support by Iso-Seq data  cat bc1008_rm.augustus.isoseq_hints.gff \| perl -ne 'if (/\ttranscript\t.*\t(\S+)/){$tx=$1;} if (/transcript supported.*100/) {print "$tx\n";}' \| tee bc1008_rm.augustus.isoseq_hints.supported.lst \| wc -l  #convert prediction results from gff to gtf  grep -P "\tAUGUSTUS\t" bc1008_rm.augustus.isoseq_hints.gff > bc1008_rm.augustus.isoseq_hints.gtf  #compute size of flanking regions  computeFlankingRegion.pl bc1008_rm.augustus.isoseq_hints.gtf > bc1008_rm.augustus.isoseq_hints.computeFlankingRegion.txt  #get supported genes and falnking regions as a gb file for training  gff2gbSmallDNA.pl --good=bc1008_rm.augustus.isoseq_hints.supported.lst bc1008_rm.augustus.isoseq_hints.gff bc1008_rm/bc1008_v152l3.bp.p_ctg.fa.masked 1079 bc1008_rm.augustus.isoseq_hints.supported.genes.gb  #get list of genes in the training gb file  cat bc1008_rm.augustus.isoseq_hints.supported.genes.gb \| perl -ne 'if(m/\/gene=\"(\S+)\"/){print "\"".$1."\"\n";}' \| sort -n > bc1008_rm.augustus.isoseq_hints.supported.genes.lst  #subset gtf file with list of genes in the training set  grep -f bc1008_rm.augustus.isoseq_hints.supported.genes.lst -F bc1008_rm.augustus.isoseq_hints.gtf > bc1008_rm.augustus.isoseq_hints.supported.genes.gtf  #get protein seqeunces of genes in the training set  gtf2aa.pl bc1008_rm/bc1008_v152l3.bp.p_ctg.fa.masked bc1008_rm.augustus.isoseq_hints.supported.genes.gtf bc1008_rm.augustus.isoseq_hints.supported.genes.faa  #blast to find none redundant proteins  aa2nonred.pl --diamond --cores=8 bc1008_rm.augustus.isoseq_hints.supported.genes.faa bc1008_rm.augustus.isoseq_hints.supported.genes.nr.faa  #get the list of none redundant genes  grep ">" bc1008_rm.augustus.isoseq_hints.supported.genes.nr.faa \| perl -pe 's/>//' > bc1008_rm.augustus.isoseq_hints.supported.genes.nr.lst  #perl get_loci_lst.pl bc1008_rm.augustus.isoseq_hints.supported.genes.gb > bc1008_rm.augustus.isoseq_hints.supported.genes.loci.lst  bash bc1008_rm.augustus.isoseq_hints.supported.get_loci_lst.sh  grep -f bc1008_rm.augustus.isoseq_hints.supported.genes.nr.lst bc1008_rm.augustus.isoseq_hints.supported.genes.loci.lst\| cut -f2 > bc1008_rm.augustus.isoseq_hints.supported.genes.loci.nr.lst  filterGenesIn.pl bc1008_rm.augustus.isoseq_hints.supported.genes.loci.nr.lst bc1008_rm.augustus.isoseq_hints.supported.genes.gb > bc1008_rm.augustus.isoseq_hints.supported.genes.nr.gb  new_species.pl --species=arabis_ciliata  etraining --species=arabis_ciliata bc1008_rm.augustus.isoseq_hints.supported.genes.nr.gb &> bc1008_rm.augustus.isoseq_hints.etraining.out  randomSplit.pl bc1008_rm.augustus.isoseq_hints.supported.genes.nr.gb 400  etraining --species=arabis_ciliata bc1008_rm.augustus.isoseq_hints.supported.genes.nr.gb.train &> bc1008_rm.augustus.isoseq_hints.etraining.train.out  tail -6 bc1008_rm.augustus.isoseq_hints.etraining.train.out \| head -3  vi /usr/local/ngseq/src/Augustus/config/species/arabis_ciliata/arabis_ciliata_parameters.cfg  augustus --species=arabis_ciliata bc1008_rm.augustus.isoseq_hints.supported.genes.nr.gb.test &> bc1008_rm.augustus.isoseq_hints.etraining.test.out  augustus --species=arabidopsis bc1008_rm.augustus.isoseq_hints.supported.genes.nr.gb.test &> bc1008_rm.augustus.isoseq_hints.etraining.arabidopsis.test.out |
| --- | --- |
| blastp | for i in bc1012.AARE bc1009.ACAE bc1008.ACIL bc1021.CHIR bc1020.CRES bc1011.NBRA bc1010.NROT  do  blastp -query $i\.pep.faa -db /srv/GT/databases/uniprot_20211022/uniprot_sprot.fasta -evalue 1e-6 -max_hsps 1 -max_target_seqs 1 -outfmt '6 std stitle' -num_threads 4 -out $i\.uniprot.blastp  sort -k1,1 -k12,12nr -k11,11n -T /scratch/qiwei/tmp/ $i\.uniprot.blastp \| sort -u -k1,1 --merge -T /scratch/qiwei/tmp/ > $i\.uniprot.blastp.top.txt  done |
| Final training (example for one species) | etraining --species=arabis_ciliata bc1008_rm.augustus.isoseq_hints.supported.genes.nr.gb.train &> bc1008_rm.augustus.isoseq_hints.etraining.final_train.out  augustus --species=arabis_ciliata bc1008_rm.augustus.isoseq_hints.supported.genes.nr.gb.test &> bc1008_rm.augustus.isoseq_hints.etraining.final_test.out |
| Functional annotation | for i in bc1012.AARE bc1009.ACAE bc1008.ACIL bc1021.CHIR bc1020.CRES bc1011.NBRA bc1010.NROT  do  perl 01_pathway.pl $i\.ipr.tsv > $i\.pathways.txt  perl 02_desc_go.pl $i\.ipr.tsv > $i\.desc_go.txt  perl 03_ontology.pl $i\.desc_go.txt > $i\.genes_annotation.txt  /usr/local/ngseq/packages/Tools/EMBOSS/6.6.0/bin/geecee $i\.cds.fna $i\.cds.gc.txt  perl /home/qiwei/perl-scripts/fas_getlen.pl $i\.cds.fna > $i\.cds.len.txt  perl 04_combine.pl $i > $i\.functional_annotation.txt  done |
| Isoseq hints augustus (example for one species) | minimap2 -ax splice -t 8 -uf --secondary=no -C5 bc1008_v152l3.bp.p_ctg.fa hq_transcripts-bc1001.fasta > bc1008_bc1001.sam  /usr/local/ngseq/src/emtrey/emtrey -m -i bc1008_bc1001.sam > bc1008_bc1001.psl  cat bc1008_bc1001.psl \| sort -s -k 14,14 \| perl -ne '@f=split; print if ($f[0]>=100)' \| blat2hints.pl --source=PB --nomult --ep_cutoff=20 --in=/dev/stdin --out=bc1008_bc1001.isoseq_hints.gff  augustus --species=arabidopsis bc1008_rm/bc1008_v152l3.bp.p_ctg.fa.masked --softmasking=on --extrinsicCfgFile=extrinsic.M.RM.PB.cfg --hintsfile=bc1008_bc1001.isoseq_hints.gff > bc1008_rm.augustus.isoseq_hints.gff 2> bc1008_rm.augustus.isoseq_hints.log & |
| Isoseq hints get loci | cat bc1008_rm.augustus.isoseq_hints.supported.genes.gb \| perl -ne '  if($_ =~ m/LOCUS\s+(\S+)\s+/){  $txLocus=$1;  }elsif($_ =~ m/\/gene=\"(\S+)\"/){  $txInGb3{$1}=$txLocus;  }  if(eof()){  foreach(keys %txInGb3){  print "$_\t$txInGb3{$_}\n";  }  } ' > bc1008_rm.augustus.isoseq_hints.supported.genes.loci.lst |
| Optimize augustus (example for one species) | optimize_augustus.pl --species=arabis_ciliata --kfold=8 bc1008_rm.augustus.isoseq_hints.supported.genes.nr.gb > bc1008_rm.augustus.isoseq_hints.optimize.out & |
| Post-processing (example for one species) | /usr/local/ngseq/src/Augustus/scripts/getAnnoFasta.pl bc1008.augustus.arabis_ciliata.gtf --seqfile=bc1008_rm/bc1008_v152l3.bp.p_ctg.fa.masked  perl /home/qiwei/perl-scripts/fas_add_anno.pl bc1008.augustus.arabis_ciliata.aa ACIL > ACIL.pep.faa  perl /home/qiwei/perl-scripts/fas_add_anno.pl bc1008.augustus.arabis_ciliata.codingseq ACIL > ACIL.cds.fna  busco -i ACIL.pep.faa -l viridiplantae_odb10 -o ACIL_busco -m prot |

Repeat annotation

| Repeatmodeler | /usr/local/ngseq/src/RepeatModeler-2.0.2a/BuildDatabase -name bc1008 bc1008_v152l3.bp.p_ctg.fa  /usr/local/ngseq/src/RepeatModeler-2.0.2a/BuildDatabase -name bc1009 bc1009_v152l3.bp.p_ctg.fa  /usr/local/ngseq/src/RepeatModeler-2.0.2a/BuildDatabase -name bc1010 bc1010_v152l3.bp.p_ctg.fa  /usr/local/ngseq/src/RepeatModeler-2.0.2a/BuildDatabase -name bc1011 bc1011_v152l3.bp.p_ctg.fa  /usr/local/ngseq/src/RepeatModeler-2.0.2a/BuildDatabase -name bc1012 bc1012_v152l3.bp.p_ctg.fa  /usr/local/ngseq/src/RepeatModeler-2.0.2a/BuildDatabase -name bc1020 bc1020_v152l3.bp.p_ctg.fa  /usr/local/ngseq/src/RepeatModeler-2.0.2a/BuildDatabase -name bc1021 bc1021_v152l3.bp.p_ctg.fa  /usr/local/ngseq/src/RepeatModeler-2.0.2a/RepeatModeler -database bc1008 -pa 4 -LTRStruct > bc1008.repeatmodeler.stdout 2> bc1008.repeatmodeler.stderr &  /usr/local/ngseq/src/RepeatModeler-2.0.2a/RepeatModeler -database bc1009 -pa 4 -LTRStruct > bc1009.repeatmodeler.stdout 2> bc1009.repeatmodeler.stderr &  /usr/local/ngseq/src/RepeatModeler-2.0.2a/RepeatModeler -database bc1010 -pa 4 -LTRStruct > bc1010.repeatmodeler.stdout 2> bc1010.repeatmodeler.stderr &  /usr/local/ngseq/src/RepeatModeler-2.0.2a/RepeatModeler -database bc1011 -pa 4 -LTRStruct > bc1011.repeatmodeler.stdout 2> bc1011.repeatmodeler.stderr &  /usr/local/ngseq/src/RepeatModeler-2.0.2a/RepeatModeler -database bc1012 -pa 4 -LTRStruct > bc1012.repeatmodeler.stdout 2> bc1012.repeatmodeler.stderr &  /usr/local/ngseq/src/RepeatModeler-2.0.2a/RepeatModeler -database bc1020 -pa 4 -LTRStruct > bc1020.repeatmodeler.stdout 2> bc1020.repeatmodeler.stderr &  /usr/local/ngseq/src/RepeatModeler-2.0.2a/RepeatModeler -database bc1021 -pa 4 -LTRStruct > bc1021.repeatmodeler.stdout 2> bc1021.repeatmodeler.stderr & |
| --- | --- |
| Repeatmasker | for i in bc1008 bc1009 bc1010 bc1011 bc1012 bc1020 bc1021  do  /usr/local/ngseq/src/RepeatMasker-4.1.2/RepeatMasker/RepeatMasker -pa 8 -lib $i\-families.fa -no_is -nolow -norna -dir $i\_rm -xsmall -html -gff -excln $i\_v152l3.bp.p_ctg.fa > $i\_rm.stdout 2> $i\_rm.stderr  done |
| Repeat landscape | perl /usr/local/ngseq/src/RepeatMasker-4.1.2/RepeatMasker/util/calcDivergenceFromAlign.pl -s bc1008_v152l3.bp.p_ctg.fa.divsum bc1008_v152l3.bp.p_ctg.fa.cat.gz  tail -n 72 bc1008_v152l3.bp.p_ctg.fa.divsum > bc1008_v152l3.bp.p_ctg.fa.Kimura.distance  perl /usr/local/ngseq/src/RepeatMasker-4.1.2/RepeatMasker/util/createRepeatLandscape.pl -div bc1008_v152l3.bp.p_ctg.fa.divsum -g 321646273 -t "bc1008 Repeat Landscape" > bc1008_v152l3.bp.p_ctg.fa.RepeatLandscape.html  perl /usr/local/ngseq/src/RepeatMasker-4.1.2/RepeatMasker/util/calcDivergenceFromAlign.pl -s bc1009_v152l3.bp.p_ctg.fa.divsum bc1009_v152l3.bp.p_ctg.fa.cat.gz &  perl /usr/local/ngseq/src/RepeatMasker-4.1.2/RepeatMasker/util/calcDivergenceFromAlign.pl -s bc1010_v152l3.bp.p_ctg.fa.divsum bc1010_v152l3.bp.p_ctg.fa.cat.gz &> alcDivergenceFromAlign.log &  perl /usr/local/ngseq/src/RepeatMasker-4.1.2/RepeatMasker/util/calcDivergenceFromAlign.pl -s bc1011_v152l3.bp.p_ctg.fa.divsum bc1011_v152l3.bp.p_ctg.fa.cat.gz &> alcDivergenceFromAlign.log &  perl /usr/local/ngseq/src/RepeatMasker-4.1.2/RepeatMasker/util/calcDivergenceFromAlign.pl -s bc1012_v152l3.bp.p_ctg.fa.divsum bc1012_v152l3.bp.p_ctg.fa.cat.gz &> alcDivergenceFromAlign.log &  perl /usr/local/ngseq/src/RepeatMasker-4.1.2/RepeatMasker/util/calcDivergenceFromAlign.pl -s bc1020_v152l3.bp.p_ctg.fa.divsum bc1020_v152l3.bp.p_ctg.fa.cat.gz &> alcDivergenceFromAlign.log &  perl /usr/local/ngseq/src/RepeatMasker-4.1.2/RepeatMasker/util/calcDivergenceFromAlign.pl -s bc1021_v152l3.bp.p_ctg.fa.divsum bc1021_v152l3.bp.p_ctg.fa.cat.gz &> alcDivergenceFromAlign.log &  tail -n 72 bc1009_v152l3.bp.p_ctg.fa.divsum > bc1009_v152l3.bp.p_ctg.fa.Kimura.distance  perl /usr/local/ngseq/src/RepeatMasker-4.1.2/RepeatMasker/util/createRepeatLandscape.pl -div bc1009_v152l3.bp.p_ctg.fa.divsum -g 397820405 -t "bc1009 Repeat Landscape" > bc1009_v152l3.bp.p_ctg.fa.RepeatLandscape.html  perl /usr/local/ngseq/src/RepeatMasker-4.1.2/RepeatMasker/util/createRepeatLandscape.pl -div bc1011_v152l3.bp.p_ctg.fa.divsum -g 268721868 -t "bc1011 Repeat Landscape" > bc1011_v152l3.bp.p_ctg.fa.RepeatLandscape.html  tail -n 72 bc1011_v152l3.bp.p_ctg.fa.divsum > bc1011_v152l3.bp.p_ctg.fa.Kimura.distance  tail -n 72 bc1010_v152l3.bp.p_ctg.fa.divsum > bc1010_v152l3.bp.p_ctg.fa.Kimura.distance  perl /usr/local/ngseq/src/RepeatMasker-4.1.2/RepeatMasker/util/createRepeatLandscape.pl -div bc1010_v152l3.bp.p_ctg.fa.divsum -g 408043968 -t "bc1010 Repeat Landscape" > bc1010_v152l3.bp.p_ctg.fa.RepeatLandscape.html  perl /usr/local/ngseq/src/RepeatMasker-4.1.2/RepeatMasker/util/createRepeatLandscape.pl -div bc1012_v152l3.bp.p_ctg.fa.divsum -g 217874282 -t "bc1012 Repeat Landscape" > bc1012_v152l3.bp.p_ctg.fa.RepeatLandscape.html  tail -n 72 bc1012_v152l3.bp.p_ctg.fa.divsum > bc1012_v152l3.bp.p_ctg.fa.Kimura.distance  tail -n 72 bc1020_v152l3.bp.p_ctg.fa.divsum > bc1020_v152l3.bp.p_ctg.fa.Kimura.distance  perl /usr/local/ngseq/src/RepeatMasker-4.1.2/RepeatMasker/util/createRepeatLandscape.pl -div bc1020_v152l3.bp.p_ctg.fa.divsum -g 245691755 -t "bc1020 Repeat Landscape" > bc1020_v152l3.bp.p_ctg.fa.RepeatLandscape.html  perl /usr/local/ngseq/src/RepeatMasker-4.1.2/RepeatMasker/util/createRepeatLandscape.pl -div bc1021_v152l3.bp.p_ctg.fa.divsum -g 208767977 -t "bc1021 Repeat Landscape" > bc1021_v152l3.bp.p_ctg.fa.RepeatLandscape.html  tail -n 72 bc1021_v152l3.bp.p_ctg.fa.divsum > bc1021_v152l3.bp.p_ctg.fa.Kimura.distance |

Orthofinder & CAFE5

| Orthofinder | orthofinder -t 32 -a 32 -M msa -S diamond -A mafft -T iqtree -f 01_transcripts/ |
| --- | --- |
| CAFE5 data preparation | #prepare orthofinder output for cafe5  awk -F'\t' '{print "(null)\t"$0}' /home/jflury/ORTHOFINDER/01_transcripts/OrthoFinder/Results_Feb02/Orthogroups/Orthogroups.GeneCount.tsv > tmp.tsv  #Change the header (null) to Desc and save  sed -i '1s/(null)/Desc/' tmp.tsv  #remove the total column from above, without needed to figure out column numbers.  awk -F'\t' '{$NF=""; print $0}' tmp.tsv \| rev \| sed 's/^\s*//g' \| rev \| tr ' ' '\t' > mod.tsv  python2.7 /share/pool/jflury/CAFE_Analysis/cafe_tutorial-main/python_scripts/cafetutorial_clade_and_size_filter.py -i mod.tsv -s -o cafe.input.tsv |
| Prepare ultrametric tree in R | tre <- read.tree("SpeciesTree_rooted.txt")  N <- Ntip(bin_tree)  root_node <- N + 1  root_to_tip <- dist.nodes(bin_tree)[1:N, root_node]  sqrt(.Machine$double.eps)  tree_ex<-bin_tree## [1] 1.490116e-08  age_diff<- max(root_to_tip) - root_to_tip  tip_edges<- tree_ex$edge[,2] <= Ntip(tree_ex)  tree_ex$edge.length[tip_edges]<- tree_ex$edge.length[tip_edges]+age_diff  is.ultrametric(bin_tree)  bin_tree<-MakeTreeBinary(tree_ex)  plot(bin_tree)  outgroup = c("A_arabicum.pro")  rooted_tree<-root(bin_tree, outgroup, edgelabel=TRUE, resolve.root=TRUE)  plot(rooted_tree)  write.tree(bin_tree, file="ultrametric_tree.tre") |
| CAFE5 | cafe5 -i cafe.input.tsv -t ultrametric_tree.tre  <https://www.arabidopsis.org/download_files/Genes/TAIR10_genome_release/TAIR10_NCBI_mapping_files/TAIR10_NCBI_REFSEQ_mapping_PROT> |
| Cafeplotter | cafeplotter -i ./results -o cafeplotter |

GO-figure

| Download data | #! /bin/bash  mkdir data  cd data  wget http://purl.obolibrary.org/obo/go/go-basic.obo  wget <ftp://ftp.ebi.ac.uk/pub/databases/GO/goa/UNIPROT/goa_uniprot_all.gaf.gz> |
| --- | --- |
| Prepare reference data | #! /bin/bash  python3 relations.py data/go-basic.obo > data/relations.tsv  cp data/relations.tsv data/relations.tab  python3 ics.py data/relations.tab data/goa_uniprot_all.gaf.gz data/go-basic.obo > data/ic.tsv |
| Prepare sample data | Modify output from here: <https://www.arabidopsis.org/tools/go_term_enrichment.jsp> to have two columns: % Goterm and enrichment_P-value |
| Run gofigure | python gofigure.py -i list_for_gofigure.txt -o output |

Repeat annotation using EarlGrey

| EarlGrey | Example:  earlGrey -g ACAE_bc1009_v152l3.bp.p_ctg.fa -s Arabis_caerulea -o ./output_more_mem2 -t 15 |
| --- | --- |

Genome size estimation using Jellyfish and GenomeScope2

| SRA-toolkit | Example:  prefetch --max-size 30G ERR3822209  fasterq-dump --split-files ERR3822209 |
| --- | --- |
| fastp | fastp -i ERR3822209_1.fastq -I ERR3822209_2.fastq -o clean_ERR3822209_1.fastq -O clean_ERR3822209_2.fastq |
| Jellyfish | jellyfish count -C -m 21 -s 100M -t 10 -o 01_jelly_fish/species_name.jf clean_ERR3822209_1.fastq clean_ERR3822209_2.fastq clean_ERR3823743_1.fastq clean_ERR3823743_2.fastq |
| GenomeScope2 | http://genomescope.org/genomescope2.0/ with default settings |

**References**

Azeez, A., & Bates, P. D. (2024). Self-incompatibility based functional genomics for rapid phenotypic characterization of seed metabolism genes. *Plant Biotechnology Journal*, *22*(10), 2688. https://doi.org/10.1111/pbi.14383

Barragan, A. C., Collenberg, M., Schwab, R., Kersten, S., Kerstens, M. H. L., Požárová, D., Bezrukov, I., Bemm, F., Kolár, F., & Weigel, D. (2024). Deleterious phenotypes in wild *Arabidopsis arenosa* populations are common and linked to runs of homozygosity. *G3 Genes|Genomes|Genetics*, *14*(3), jkad290. https://doi.org/10.1093/g3journal/jkad290

Bateman, A. J. (1955). Self-incompatibility systems in angiosperms: III. Cruciferae. *Heredity*, *9*(1), 53–68. https://doi.org/10.1038/hdy.1955.2

Boaz, M., Plitmann, U., & Heyn, C. C. (1990). The ecogeographic distribution of breeding systems in the cruciferae (Brassicaceae) of Israel. *Israel Journal of Plant Sciences*, *39*(1–2), 31–42. https://doi.org/10.1080/0021213X.1990.10677132

Bowman, J. L., Brüggemann, H., Lee, J., & Mummenhoff, K. (1999). Evolutionary changes in floral structure within *Lepidium L.* (Brassicaceae). *International Journal of Plant Sciences*, *160*(5), 917–929. https://doi.org/10.1086/314194

Busch, J. W. (2005). Inbreeding depression in self-incompatible and self-compatible populations of *Leavenworthia alabamica*. *Heredity*, *94*(2), 159–165. https://doi.org/10.1038/sj.hdy.6800584

Byrne, S. L., Erthmann, P. Ø., Agerbirk, N., Bak, S., Hauser, T. P., Nagy, I., Paina, C., & Asp, T. (2017). The genome sequence of *Barbarea vulgaris* facilitates the study of ecological biochemistry. *Scientific Reports*, *7*(1), 40728. https://doi.org/10.1038/srep40728

Christensen, S., Sørensen, H., Munk, K. R., & Hauser, T. P. (2016). A hybridisation barrier between two evolutionary lineages of *Barbarea vulgaris* (Brassicaceae) that differ in biotic resistances. *Evolutionary Ecology*, *30*(5), 887–904. https://doi.org/10.1007/s10682-016-9858-z

Danin, A., & Feinbrun-Dothan, N. (1991). *Analytical Flora of Eretz-Israel.* CANA Publishing House Ltd.

Dassanayake, M., Oh, D.-H., Haas, J. S., Hernandez, A., Hong, H., Ali, S., Yun, D.-J., Bressan, R. A., Zhu, J.-K., Bohnert, H. J., & Cheeseman, J. M. (2011). The genome of the extremophile crucifer *Thellungiella parvula*. *Nature Genetics*, *43*(9), Article 9. https://doi.org/10.1038/ng.889

Guo, X., Hu, Q., Hao, G., Wang, X., Zhang, D., Ma, T., & Liu, J. (2018). The genomes of two *Eutrema* species provide insight into plant adaptation to high altitudes. *DNA Research*, *25*(3), 307–315. https://doi.org/10.1093/dnares/dsy003

Haudry, A., Platts, A. E., Vello, E., Hoen, D. R., Leclercq, M., Williamson, R. J., Forczek, E., Joly-Lopez, Z., Steffen, J. G., Hazzouri, K. M., Dewar, K., Stinchcombe, J. R., Schoen, D. J., Wang, X., Schmutz, J., Town, C. D., Edger, P. P., Pires, J. C., Schumaker, K. S., … Blanchette, M. (2013). An atlas of over 90,000 conserved noncoding sequences provides insight into crucifer regulatory regions. *Nature Genetics*, *45*(8), 891–898. https://doi.org/10.1038/ng.2684

Hickey, M., & Clive, J. (1988). *100 families of flowering plants* (2d ed.). Cambridge University Press.

Hu, T. T., Pattyn, P., Bakker, E. G., Cao, J., Cheng, J.-F., Clark, R. M., Fahlgren, N., Fawcett, J. A., Grimwood, J., Gundlach, H., Haberer, G., Hollister, J. D., Ossowski, S., Ottilar, R. P., Salamov, A. A., Schneeberger, K., Spannagl, M., Wang, X., Yang, L., … Guo, Y.-L. (2011). The *Arabidopsis lyrata* genome sequence and the basis of rapid genome size change. *Nature Genetics*, *43*(5), 476–481. https://doi.org/10.1038/ng.807

Jiao, W.-B., Accinelli, G. G., Hartwig, B., Kiefer, C., Baker, D., Severing, E., Willing, E.-M., Piednoel, M., Woetzel, S., Madrid-Herrero, E., Huettel, B., Hümann, U., Reinhard, R., Koch, M. A., Swan, D., Clavijo, B., Coupland, G., & Schneeberger, K. (2017). Improving and correcting the contiguity of long-read genome assemblies of three plant species using optical mapping and chromosome conformation capture data. *Genome Research*, *27*(5), 778–786. https://doi.org/10.1101/gr.213652.116

Johnston, C. R., Horn, P. J., & Alonso, A. P. (2024). First draft reference genome and annotation of the alternative oil species *Physaria fendleri*. *G3: Genes | Genomes | Genetics*, *14*(9), jkae114. https://doi.org/10.1093/g3journal/jkae114

Kiefer, C., Willing, E.-M., Jiao, W.-B., Sun, H., Piednoël, M., Hümann, U., Hartwig, B., Koch, M. A., & Schneeberger, K. (2019). Interspecies association mapping links reduced CG to TG substitution rates to the loss of gene-body methylation. *Nature Plants*, *5*(8), 846–855. https://doi.org/10.1038/s41477-019-0486-9

Kliver, S., Rayko, M., Komissarov, A., Bakin, E., Zhernakova, D., Prasad, K., Rushworth, C., Baskar, R., Smetanin, D., Schmutz, J., Rokhsar, D. S., Mitchell-Olds, T., Grossniklaus, U., & Brukhin, V. (2018). Assembly of the *Boechera retrofracta* genome and evolutionary analysis of apomixis-associated genes. *Genes*, *9*(4), 185. https://doi.org/10.3390/genes9040185

Kropf, M., Comes, H. P., & Kadereit, J. W. (2006). Long-distance dispersal vs vicariance: The origin and genetic diversity of alpine plants in the Spanish Sierra Nevada. *New Phytologist*, *172*(1), 169–184. https://doi.org/10.1111/j.1469-8137.2006.01795.x

Kudoh, H. (2016). Molecular phenology in plants: In natura systems biology for the comprehensive understanding of seasonal responses under natural environments. *New Phytologist*, *210*(2), 399–412. https://doi.org/10.1111/nph.13733

Laenen, B., Tedder, A., Nowak, M. D., Toräng, P., Wunder, J., Wötzel, S., Steige, K. A., Kourmpetis, Y., Odong, T., Drouzas, A. D., Bink, M. C. A. M., Ågren, J., Coupland, G., & Slotte, T. (2018). Demography and mating system shape the genome-wide impact of purifying selection in *Arabis alpina*. *Proceedings of the National Academy of Sciences*, *115*(4), 816–821. https://doi.org/10.1073/pnas.1707492115

Mishra, B., Ploch, S., Runge, F., Schmuker, A., Xia, X., Gupta, D. K., Sharma, R., & Thines, M. (2020). The genome of *Microthlaspi erraticum* (brassicaceae) provides insights into the adaptation to highly calcareous soils. *Frontiers in Plant Science*, *11*. https://doi.org/10.3389/fpls.2020.00943

Mitchell, R. J. (1997). Effects of pollination intensity on *Lesquerella fendleri* seed set: Variation among plants. *Oecologia*, *109*(3), 382–388. https://doi.org/10.1007/s004420050097

Olowokudejo, J. D., & Heywood, V. H. (1984). Cytotaxonomy and breeding system of the genus *Biscutella* (Cruciferae). *Plant Systematics and Evolution*, *145*(3), 291–309. https://doi.org/10.1007/BF00983955

Rushworth, C. A., Song, B.-H., Lee, C.-R., & Mitchell-Olds, T. (2011). *Boechera*, a model system for ecological genomics. *Molecular Ecology*, *20*(23), 4843–4857. https://doi.org/10.1111/j.1365-294X.2011.05340.x

Sailer, C., Babst-Kostecka, A., Fischer, M. C., Zoller, S., Widmer, A., Vollenweider, P., Gugerli, F., & Rellstab, C. (2018). Transmembrane transport and stress response genes play an important role in adaptation of *Arabidopsis halleri* to metalliferous soils. *Scientific Reports*, *8*, 16085. https://doi.org/10.1038/s41598-018-33938-2

Schmickl, R., Jørgensen, M. H., Brysting, A. K., & Koch, M. A. (2010). The evolutionary history of the *Arabidopsis lyrata* complex: A hybrid in the amphi-Beringian area closes a large distribution gap and builds up a genetic barrier. *BMC Evolutionary Biology*, *10*(1), 98. https://doi.org/10.1186/1471-2148-10-98

Slotte, T., Hazzouri, K. M., Ågren, J. A., Koenig, D., Maumus, F., Guo, Y.-L., Steige, K., Platts, A. E., Escobar, J. S., Newman, L. K., Wang, W., Mandáková, T., Vello, E., Smith, L. M., Henz, S. R., Steffen, J., Takuno, S., Brandvain, Y., Coop, G., … Wright, S. I. (2013). The *Capsella rubella* genome and the genomic consequences of rapid mating system evolution. *Nature Genetics*, *45*(7), 831–835. https://doi.org/10.1038/ng.2669

Theologis, A., Ecker, J. R., Palm, C. J., Federspiel, N. A., Kaul, S., White, O., Alonso, J., Altafi, H., Araujo, R., Bowman, C. L., Brooks, S. Y., Buehler, E., Chan, A., Chao, Q., Chen, H., Cheuk, R. F., Chin, C. W., Chung, M. K., Conn, L., … Davis, R. W. (2000). Sequence and analysis of chromosome 1 of the plant *Arabidopsis thaliana*. *Nature*, *408*(6814), 816–820. https://doi.org/10.1038/35048500

Yang, R., Jarvis, D. J., Chen, H., Beilstein, M., Grimwood, J., Jenkins, J., Shu, S., Prochnik, S., Xin, M., Ma, C., Schmutz, J., Wing, R. A., Mitchell-Olds, T., Schumaker, K., & Wang, X. (2013). The reference genome of the halophytic plant *Eutrema salsugineum*. *Frontiers in Plant Science*, *4*. https://www.frontiersin.org/journals/plant-science/articles/10.3389/fpls.2013.00046

Zhang, T., Qiao, Q., Novikova, P. Yu., Wang, Q., Yue, J., Guan, Y., Ming, S., Liu, T., De, J., Liu, Y., Al-Shehbaz, I. A., Sun, H., Van Montagu, M., Huang, J., Van De Peer, Y., & Qiong, L. (2019). Genome of *Crucihimalaya himalaica* , a close relative of *Arabidopsis* , shows ecological adaptation to high altitude. *Proceedings of the National Academy of Sciences*, *116*(14), 7137–7146. https://doi.org/10.1073/pnas.1817580116
